# Decreased skin colonization with *Malassezia* spp. and increased skin colonization with *Candida* spp. in patients with severe atopic dermatitis

**DOI:** 10.3389/fmed.2024.1353784

**Published:** 2024-02-20

**Authors:** Lukas Storz, Bettina Schmid, Philipp Peter Bosshard, Peter Schmid-Grendelmeier, Marie-Charlotte Brüggen, Claudia Lang

**Affiliations:** ^1^Department of Dermatology, University Hospital Zurich, Zurich, Switzerland; ^2^Faculty of Medicine, University of Zurich, Zurich, Switzerland; ^3^Medical Campus Davos, Davos, Switzerland

**Keywords:** atopic dermatitis, *Malassezia* spp., *Candida* spp., sensitization patterns, skin microbiota

## Abstract

**Background:**

Atopic dermatitis (AD) is a chronic relapsing inflammatory skin disease in which patients are sensitized towards a plethora of allergens. The hosts fungal microbiota, the mycobiota, that is believed to be altered in patients suffering from AD acts as such an allergen. The correlation context of specific sensitization, changes in mycobiota and its impact on disease severity however remains poorly understood.

**Objectives:**

We aim to enhance the understanding of the specific sensitization towards the mycobiota in AD patients in relation to their fungal skin colonization.

**Methods:**

Sensitization pattern towards the *Malassezia* spp. and *Candida albicans* of 16 AD patients and 14 healthy controls (HC) were analyzed with the newly developed multiplex-assay ALEX^2®^ and the established singleplex-assay ImmunoCAP^®^. We compared these findings with the fungal skin colonization analyzed by DNA sequencing of the internal transcribed spacer region 1 (ITS1).

**Results:**

Sensitization in general and towards *Malassezia* spp. and *C. albicans* is increased in AD patients compared to HC with a quantitative difference in severe AD when compared to mild to moderate AD. Further we saw an association between sensitization towards and skin colonization with *Candida* spp. yet a negative correlation between sensitization towards and skin colonization with *Malassezia* spp.

**Conclusion:**

We conclude that AD in general and severe AD in particular is associated with increased sensitization towards the hosts own mycobiota. There is positive correlation in *Candida* spp. skin colonization and negative in *Malassezia* spp. skin colonization when compared to AD, AD severity as well as to specific sensitization patterns.

## Introduction

Atopic dermatitis (AD) is a chronic relapsing inflammatory skin disease. It is characterized by age-dependent typical eczematous lesions, xerosis, pruritus with a chronic disease course and is often associated with allergic rhino conjunctivitis and asthma (1). In most cases onset occurs in early childhood from 3 months to 2 years, yet a substantial portion of these patients go into complete remission before the age of 2 years (2). AD has experienced an epidemic rise, i.e., a three-fold increase over the past 30 years. It now affects 15% to 30% of all children and 2 to10% of the adults (1). Having the highest burden of Disability-adjusted life years (DALY) of all skin diseases (3) AD has an important health effect at population level (4).

Common risk factors of developing AD are mutations in genes encoding for structural proteins such as the loss-of-function mutation within the FLG-gene encoding for filaggrin, epidermal proteases and protease inhibitors (5, 6). The strongest risk factor overall is a positive family history for atopic diseases with active AD in particular (7). Further, environmental properties such as small family size, living in urban settings and regions with low exposure to UV radiation, low humidity and western-diet predispose to AD (4).

With these structural and environmental hazards, the pathophysiology is not yet fully explained. It is widely accepted as a pathophysiological concept that AD evolves due to an interplay of a genetic background, impaired skin barrier as well as an aberrant skin immune response (8, 9). On one hand, the barrier dysfunction allows substances like bacteria or allergens from the environment to penetrate the skin. On the other hand, these substances can stimulate keratinocytes to produce a variety of cytokines and thus trigger a Th2-mediated immune response (1, 10). These environmental particles include the host’s fungal and bacterial skin microbiota (11).

The role of *Staphylococcus aureus*, a pathogenic bacteria that colonizes human skin with a much higher burden in AD patients than in healthy controls (HC) (12, 13) and acts as a driver of inflammatory skin eruption through immune modulation, is already well studied (14, 15). In contrast studies describing the host’s fungal microbiota (mycobiota) are rare (16, 17). Two of the most abundant fungal microorganisms on human skin are *Malassezia* spp. and *Candida* spp. (18, 19).

*Malassezia* is a lipid-dependent yeast and is by far the most abundant fungus found on Caucasian skin (19, 20). It currently includes 14 species, among which 9 species colonize human and 5 animal skin (21). Within the human colonizing species, there are differences in Th2-dependent immune response found in human keratinocytes and dendritic cells. Dependent on the species the immune pattern shows elevated levels of pro-inflammatory cytokines such as IL-4, IL-13 and IL-17 which compromise the skin’s innate immune response towards microorganisms (17, 22). The microenvironment shows susceptibility for secondary infections on top of amplifying specific AD inflammation patterns (23). While sensitization towards *Malassezia* spp. has been shown to be an important allergen-specific marker for AD severity in Caucasian adults (24, 25), the impact of the amount of fungal colonization in people suffering from AD remains unclear (25–27).

Another commonly detected yeast on human skin is *Candida albicans* (*C. albicans*), which contains several antigenic components capable of stimulating immediate hypersensitivity responses and sensitization towards its allergens and thus contributes to the pathogenesis of AD (11, 28). *C. albicans* burden seems higher in AD patients compared to HC, yet effects on pathomechanism and disease progression remain unclear (29, 30).

Treatment of AD includes pharmacological and non-pharmacological therapy and should be applied depending on clinical severity an individual’s phenotype in a stepwise manner (31). Standard pharmacological therapy comprises of basic skin care with emollients, topical anti-inflammatory therapy with corticosteroids or calcineurin inhibitors and eventually systemic therapy with short term corticosteroids, ciclosporin, biologicals and Janus kinase inhibitors (31–33). Non-pharmacological treatment includes phototherapy, stress management, psychoeducation and identification and reduction of AD triggers and (31). AD patients with head and neck phenotypes, as well as patients with IgE sensitization towards *Malassezia* spp. (34) may benefit from antifungal therapy, although additional studies to properly identify beneficial patient characteristics are needed (35). Azole derivates such as ketoconazole and itraconazole are among the most studied antifungal therapies and can be applied topically or systemically (31, 36).

The aim of this study was to examine differences in sensitization towards *Malassezia* spp. and *C. albicans* between AD patients and HC and to put it in perspective to the skin mycobiome and to the impact on disease severity.

## Methods

### Study design

We included 16 AD patients from the Prospective Longitudinal Observational Research in Atopic Dermatitis (ProRaD)-Cohort who were recruited from March 2018 to December 2019 and treated at the University hospital Zurich, Switzerland. Patients being older than 18 years, suffering from atopic dermatitis and who provided written informed consent were eligible to participate. Before enrolment, the clinical diagnosis of AD had to be confirmed by a dermatologist at the Department of Dermatology, University Hospital Zurich, Switzerland, based on the criteria of Hanifin and Rajka (37). Exclusion criteria were refusal to participate on the study or being unable to give consent.

As an age-matched control group, we included 14 healthy controls (HC). Inclusion criteria were age above 18 years, no history of AD or any other skin disease, negative skin prick test (SPT) to common allergens, no chronic medical condition or treatment and neither used antifungals nor antibiotics within 6 months prior to sampling. Exclusion criteria were refusal to participate on the study or being unable to give consent.

This study was approved by the local ethics committee (EK 2016-00301) and conducted according to the Declaration of Helsinki. All study subjects participated voluntarily and gave written informed consent.

### AD clinical assessment

All AD patients underwent physical examination by a dermatologist at the Department of Dermatology, University Hospital Zurich, Switzerland. To assess disease severity, the SCORing Atopic Dermatitis (SCORAD) index (38) was used. Classification was made in mild, moderate or severe AD with a SCORAD of <25, 25–50 or >50 (with a maximum score of 103) respectively (39).

### Multiple allergen testing with ALEX^2®^

Blood was withdrawn from all study participants, processed immediately after collection and stored at −80°C until further processing. For ALEX^2®^ protocol, the samples were analyzed and calibrated according to the manufacturer’s instructions (Allergy Explorer-ALEX^2®^ Macro-Array DX Wien, Austria) for the presence of total IgE (tIgE) and specific IgE (sIgE) antibodies using ALEX^2®^, a colorimetric enzyme multiplex assay. The test contained 183 components and 117 allergen-extracts (Supplementary Table S1). Concentrations of sIgE and tIgE were measured and classified in 5 levels (0–4 corresponding to <0.3 kU_A_/l, 0.3–1 kU_A_/l, 1–5 kU_A_/L, 5–15 kU_A_/l and >15 kU_A_/l).

### ImmunoCAP^®^

An ImmunoCAP^®^ (Thermo Fisher Scientific, Massachusetts, United States) assay was used to confirm total sensitization, sensitization to *Malassezia* spp. antigen and to further investigate specific sensitization towards *C. albicans*. The samples were analyzed and calibrated according to the manufacturer’s instructions for the presence of tIgE and sIgE using whole allergen extract m227 and m5. Concentrations of sIgE and tIgE were measured and classified in 7 levels (0–6 corresponding to <0.35 kU_A_/l, 0.35–0.7 kU_A_/l, 0.7–3.5 kU_A_/l, 3.5–17.5 kU_A_/l, 17.5–50 kU_A_/l, 50–100 kU_A_/l and >100 kU_A_/l).

### Mycobiome sampling, DNA extraction, amplification, sequencing and taxonomic classification

The fungal skin mycobiota was assessed as in Schmid et al. (40). Shortly, four common eczema skin sites in adults (antecubital crease, dorsal neck, glabella, and vertex) were swabbed with Floqswabs or eSwabs (COPAN, Brescia, Italy) soaked in sterile NaCl (0.9%, Braun, Sempach, Switzerland) by repeatedly rubbing 4–8 cm^2^ of skin surface on each skin site. As negative controls, swabs without skin contact and water were used.

DNA extraction was performed by adding extraction buffer [1 M Tris-HCl (pH 8), 50 mM EDTA (Thermo Fisher Scientific, Rheinach, Switzerland) and 0.5% Tween 20 (Bio-Rad Laboratories, Cressier, Switzerland)] with proteinase K (Roche Diagnostics, Rotkreuz, Switzerland) to the sample and overnight pre-digestion at 56°C. The following day, the cell walls were mechanically disrupted with 0.5 mm beads (Qiagen, Hilden, Germany) in a Tissuelyser (Qiagen) and further with the Masterpure Yeast DNA Purification kit (Epicentre, LuBioScience GmbH, Zurich, Switzerland) according to the manufacturer’s protocol.

PCR amplification of the internal transcribed spacer region 1 (ITS1) region was performed with the primers 18S-F (5′-GTAAAAGTCGTAACAAGGTTTC-3′) and 5.8S-1R (5′-GTTCAAAGAYTCGATGATTCAC-3′) (19). PCR was executed with 1 to 5 μL DNA and the Kapa Hifi Hotstart polymerase (Roche Diagnostics). Cycling conditions were as follows: pre-incubation at 95°C for 300 s, 33 cycles of 98°C for 20 s, 61°C for 20 s, 72°C for 40 s, and final extension at 72°C for 60 s. Libraries were created following the guidelines of Illumina’s 16S rRNA metagenomics sequencing library preparation, incorporating certain modifications (41). Shortly, the DNA amplicon was evaluated on a 1.5% agarose gel after the first PCR before adding Nextera XT Indices (Illumina, San Diego, CA, United States) by a second PCR with 8 cycles. Agencourt Ampure XP beads (Beckman Coulter, Krefeld, Germany) were used for purification. Subsequently PCR amplicons were quantified with a Qubit 2.0 Fluorometer (Thermo Fisher Scientific), normalized to 4 nM, and pooled. Libraries were finalized with the Miseq Regent kit v3 (Illumina) and 2 × 300 pb paired-end reads were generated with the Miseq sequencing machine (Illumina).

Raw ITS reads were processed with PIPITS (v2.3) (42) with default parameters and the RDP Classifier (v2.1211) (43) against UNITE database (04.02.2020) (44) to generate taxonomic classification. BLAST was used for manual adjustment of the taxonomy table for interesting sequences with >1,000 contigs and >97% identity (45).

### Data analysis and statistics

For data analysis and statistics for interpretation of baseline characteristics and sensitization patterns IBM^®^ SPSS ^®^ Statistics for Windows, version 28 (IBM Corp., Armonk, NY, United States) was used. Nonparametric statistical analysis was performed using Kruskal–Wallis test for multiple comparisons among the whole study-population with post-hoc Mann–Whitney-*U* test to further investigate pairwise subgroup differences. *p*-values <0.05 were considered as statistically significant. The effect size of Mann–Whitney-*U* test was calculated using Pearson’s correlation coefficient, where values <0.3 were considered small, between 0.3–0.5 medium and >0.5 large using Cohen’s classification. Correlation analysis was performed using Spearman’s rank-order correlation (*R*), with two-sided *p*-values of <0.05 considered as statistically significant.

Data analysis of the ITS sequencing data was performed with the R software (46) by using following packages: phyloseq (47), ape (48), and tidyverse (49). For decontamination, the R package decontam was used by applying the prevalence method with a threshold of 0 (50).

## Results

### Patient characteristics

A total of 16 patients diagnosed with AD were included in this study, with 9 patients classified as mild to moderate AD and 7 patients as severe AD. 10 (63%) of these 16 patients were males. The median age for all patients was 31 years, ranging from 18 to 65 years. Five (31%) patients had head and neck type AD and median onset of AD was 1 year, ranging from 0 to 38 years. 9 (56%) patients had history of asthma, 14 (88%) patients had history of allergies. Family history for AD was positive in 7 patients (44%). Fourteen patients (88%) used topical steroids during the last 6 months while only 2 (13%) used topical antimycotics (Table 1).

**Table 1:** Characteristics of study population according to disease-severity including demographic information, disease background and medication.

	Healthy controls	Atopic dermatitis		
		Total	Mild to moderate AD	Severe AD
*Characteristic*				
Subjects, *N*	14	16	9	7
Sex, males (%)	5 (36)	10 (63)	4 (44)	6 (86)
Age, median (range)	30 (22–63)	31 (18–65)	23 (18–65)	36 (24–63)
	**Disease background**			
HNAD, *n* (%)	—	5 (31)	3 (33)	2 (29)
SCORAD, median (range)	—	45,2 (14.4–76.1)	38.3 (14.4–45.2)	68.2 (50.9–76.1)
EASI, median (range)	—	17.1 (1–34)	5.7 (1.2–18)	23.3 (16.9–23.3)
DLQI, median (range)	—	6 (1–27)	5 (1–9)	7 (3–27)
AD onset, y median (range)	—	1 (0–38)	3 (0–38)	0 (0–9)
*Personal history of*				
Asthma, *n* (%)	0 (0)	9 (56)	4 (44)	5 (71)
Allergies, *n* (%)	0 (0)	14 (88)	8 (89)	6 (86)
Positive SPT, *N* (%)	0 (0)	12 (75)	6 (67)	6 (85)
*Family history of*				
AD, *n* (%)	0 (0)	7 (44)	3 (33)	4 (57)
Asthma, *n* (%)	1 (7)	6 (38)	3 (33)	3 (43)
Allergies, *n* (%)	6 (43)	11 (69)	5 (56)	6 (86)
Smoker, *n* (%)	3 (21)	5 (31)	1 (11)	4 (57)
	**Medication during last 6 months**			
*Top. steroid*				
Class I, *n* (%)	0 (0)	0 (0)	0 (0)	0 (0)
Class II, *n* (%)	0 (0)	3 (19)	1 (11)	2 (29)
Class III, *n* (%)	0 (0)	12 (75)	6 (67)	6 (86)
Class VI, *n* (%)	0 (0)	2 (13)	0 (0)	2 (29)
Syst. steroids, *n* (%)	0 (0)	5 (31)	3 (33)	2 (29)
Dupilumab, *n* (%)	0 (0)	6 (38)	1 (11)	5 (71)
Ketoconazole, *n* (%)	0 (0)	2 (13)	1 (11)	1 (14)
TCI, *n* (%)	0 (0)	4 (25)	1 (11)	3 (43)

AD, atopic dermatitis; HNAD, head and neck type atopic dermatitis; SCORAD, SCORing Atopic Dermatitis; EASI, eczema area and severity index; DLQI, dermatology life quality index; SPT, skin prick test; Top., topical; Syst., systemic; TCI, topical calcineurin inhibitor.

### Gradual increase in tIgE and sensitization towards yeast antigens from HC to severe AD patients

**Figure 1:**
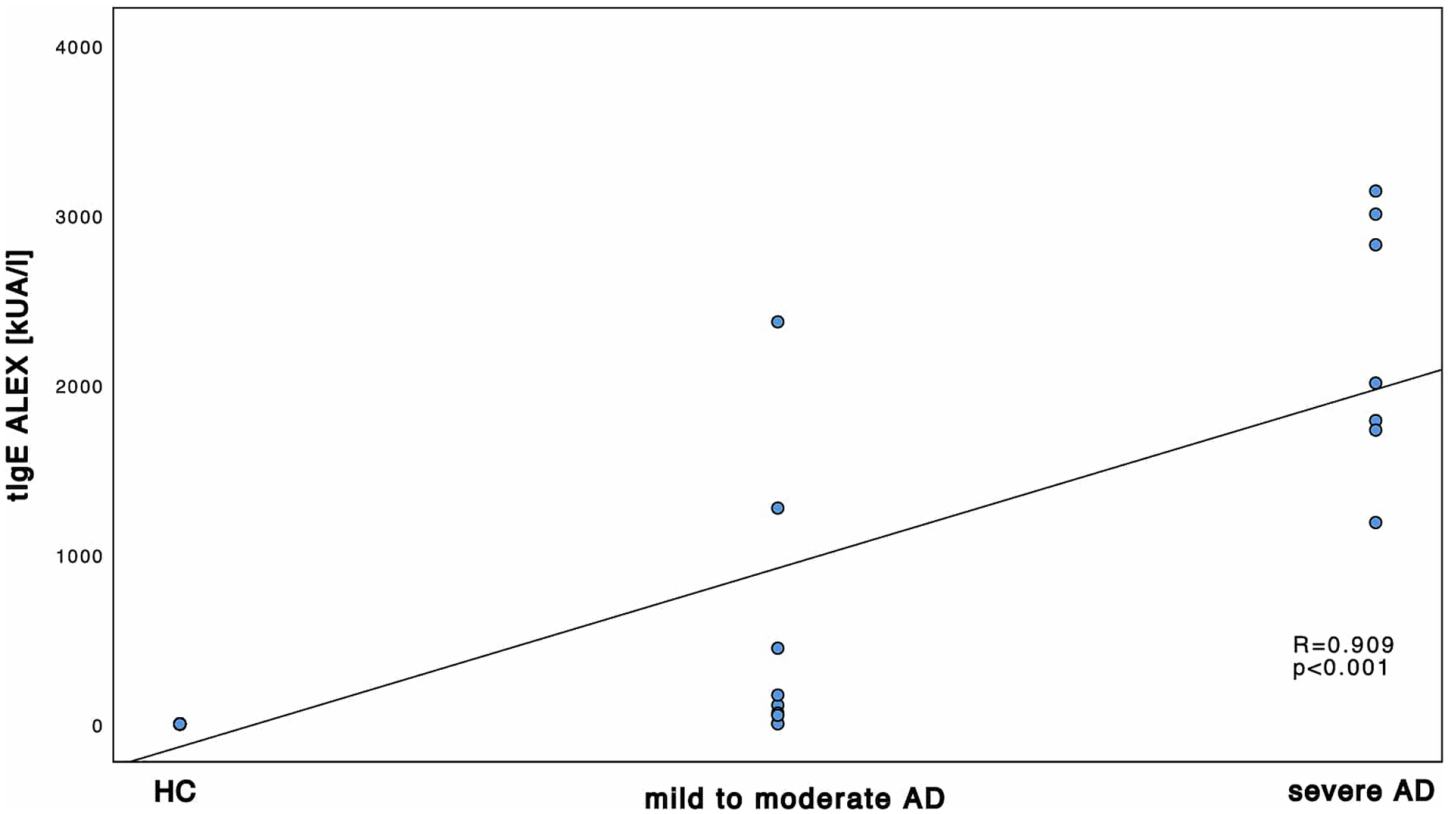
Correlation of tIgE and disease severity. tIgE using and ALEX^2®^. tIgE is measured in kU_A_/l and shown in correlation to the 3 subgroups HC, mild to moderate AD and severe AD. Results of Spearman’s correlations are illustrated in form of *R* and *p*-values.

First, we investigated the total amount of tIgE in all 16 AD patients and 14 HC. There was a significant difference between HC and AD patients altogether as well as between HC and mild to moderate and severe AD, respectively. The differences were significant both in ALEX^2®^ (Supplementary Table S2; Mann–Whitney-*U* test, *p* < 0.001, Cohen’s effect size *r* = 0.84 and *r* = 0.77 respectively) and ImmunoCAP^®^ (Supplementary Table S2; Mann–Whitney-*U* test, *p* = 0.002 and *p* < 0.001 respectively, Cohen’s effect size *r* = 0.66 and *r* = 0.77 respectively). tIgE levels did not significantly differ between mild to moderate AD and severe AD. Further, positive and statistically significant correlation with large effect size was found between disease severity and tIgE among both protocols of ImmunoCAP^®^ and ALEX^2®^ (Figure 1).

**Figure 2:**
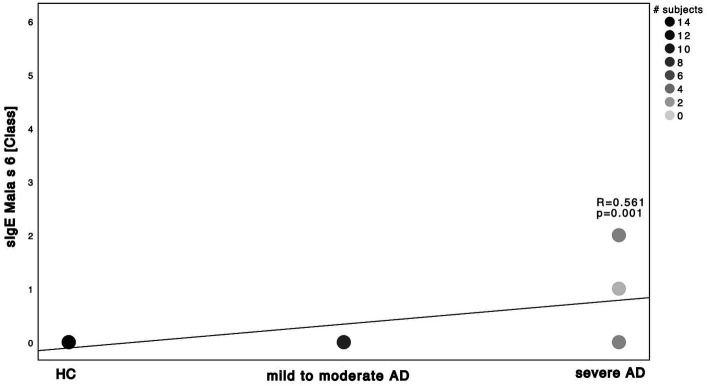
Spearman correlations of *M. sympodialis* ALEX^2®^ and disease severity. sIgE using ALEX^2®^ and displayed as class in correlation to the 3 subgroups HC, mild to moderate AD and severe AD. Darker dots indicate larger number of subjects. Results of Spearman’s correlation are illustrated in form of *R* and *p*-values.

**Figure 3:**
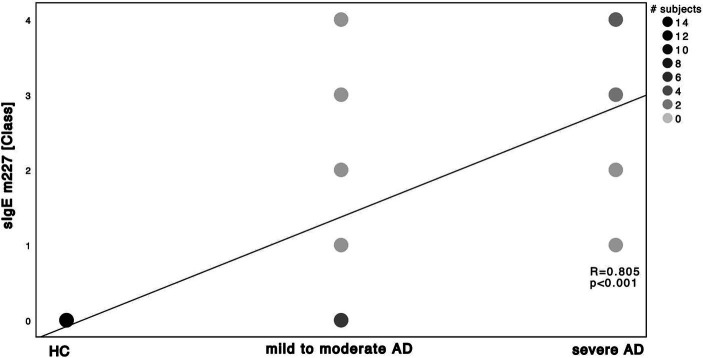
Spearman’s correlation of *Malassezia* spp. ImmunoCAP^®^ and disease severity. sIgE using ImmunoCAP^®^ and displayed as ImmunoCAP^®^ class in correlation to the 3 subgroups HC, mild to moderate AD and severe AD. m227 displays allergens of *M. sympodialis*, *M. globosa* and *M. restricta*. Darker dots indicate larger number of subjects. Results of Spearman’s correlation are illustrated in form of *R* and *p*-values.

**Figure 4:**
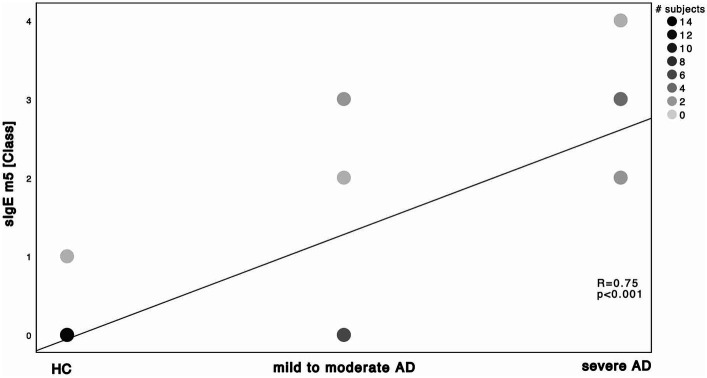
Spearman’s correlation of *C. albicans* ImmunoCAP^®^ and disease severity. sIgE m5 using ImmunoCAP^®^ and displayed as ImmunoCAP^®^ class in correlation to the 3 subgroups HC, mild to moderate AD and severe AD. Darker dots indicate larger number of subjects. Results of Spearman’s correlation are illustrated in form of *R* and *p*-values.

Next, we investigated the specific sensitization patterns towards *Malassezia* spp., *C. albicans* and further molds (*Alternaria alternata*, *Aspergillus fumigatus*, *Cladosporium herbarum*, *Penicilium chrysogenum*) present in the ALEX^2®^ macroarray. There was a significant difference in *M. sympodialis* Mala s 6 sIgE (ALEX^2®^) as well as *C. albicans* m5 sIgE (ImmunoCAP^®^) between HC and severe AD patients as well as between mild to moderate AD and severe AD (Supplementary Table S2; Mann–Whitney-*U* test, *p* = 0.002, *p* = 0.01 with Cohen’s effect size *r* = 0.67, *r* = 0.63, respectively for Mala s 6. *p* = <0.001, *p* = 0.63 with Cohen’s effect size *r* = 0.96, *r* = 0.63, respectively for m5). Further we saw a significant difference of *Malassezia* spp. m227 (ImmunoCAP^®^) between HC and mild to moderate AD as well as between mild to moderate AD to severe AD (Supplementary Table S2; Mann–Whitney-*U* test, *p* = 0.01 and *p* = 0.02 respectively, Cohen’s effect size *r* = 0.78 and *r* = 0.57 respectively). There was only one subject of HC that showed a positive yet weak sensitization towards *C. albicans* m5 with no other positive sensitization towards the other fungal antigens neither in ALEX^2®^ nor in ImmunoCAP^®^ among all HC. When looking at correlation between disease severity and sensitization pattern, we found a positive, statistically significant correlation with large effect size in *M. sympodialis* Mala s 6 using ALEX^2®^, *Malassezia* spp. m227 and *C. albicans* m5 using ImmunoCAP^®^ respectively with increasing disease severity (Figures 2–4). Between disease severity and the other two *M. sympodialis* components (Mala s 5 & Mala s 11) in ALEX^2®^, we saw a positive, statistically significant correlation with medium effect size in Mala s 5 (*R* = 0.366, *p*-value = 0.046) and Mala s 11 (*R* = 0.374, *p*-value = 0.042) respectively.

Concerning further molds in the ALEX^2®^ macroarray, only *Penicilium chrysogenum* showed a significant difference between HC and severe AD as well as between mild to moderate AD to severe AD (Supplementary Table S2).

### Increased sIgE levels against common inhalative and food allergens in AD vs. HC

Next, to tIgE and fungal sIgE levels, the ALEX^2®^ macroarray measures sIgE against a series of allergens such as tree-, grass and weed pollen, dander & epithelia, cockroaches & mites, molds & yeasts as well as food. Being sensitized towards *M. sympodialis* highly correlates with sensitization towards these common inhalative and food allergens among our study population (Supplementary Table S3). Further, there is a specific sensitization in HC (Supplementary Figure S1) with polysensitization in AD study population (Supplementary Figures S2, S3 respectively).

### Comparison between ImmunoCAP^®^ and ALEX^2®^ protocol

Comparing the two methods to detect tIgE and sIgE, we saw a positive, statistically significant and strong correlation between tIgE ImmunoCAP^®^ and ALEX^2®^ (Supplementary Figure S4). Looking at the *Malassezia* spp. and *M. sympodialis* extracts and components in ImmunoCAP^®^ and ALEX^2®^, we only saw medium effect size yet statistically significant correlation between m227 sIgE and Mala s 5 & 6 sIgE, respectively, with weak effect size and no significant correlation between m227 sIgE and Mala s 11 sIgE (Supplementary Figure S5).

### Decrease in *Malassezia* spp. colonization in severe AD

**Figure 5:**
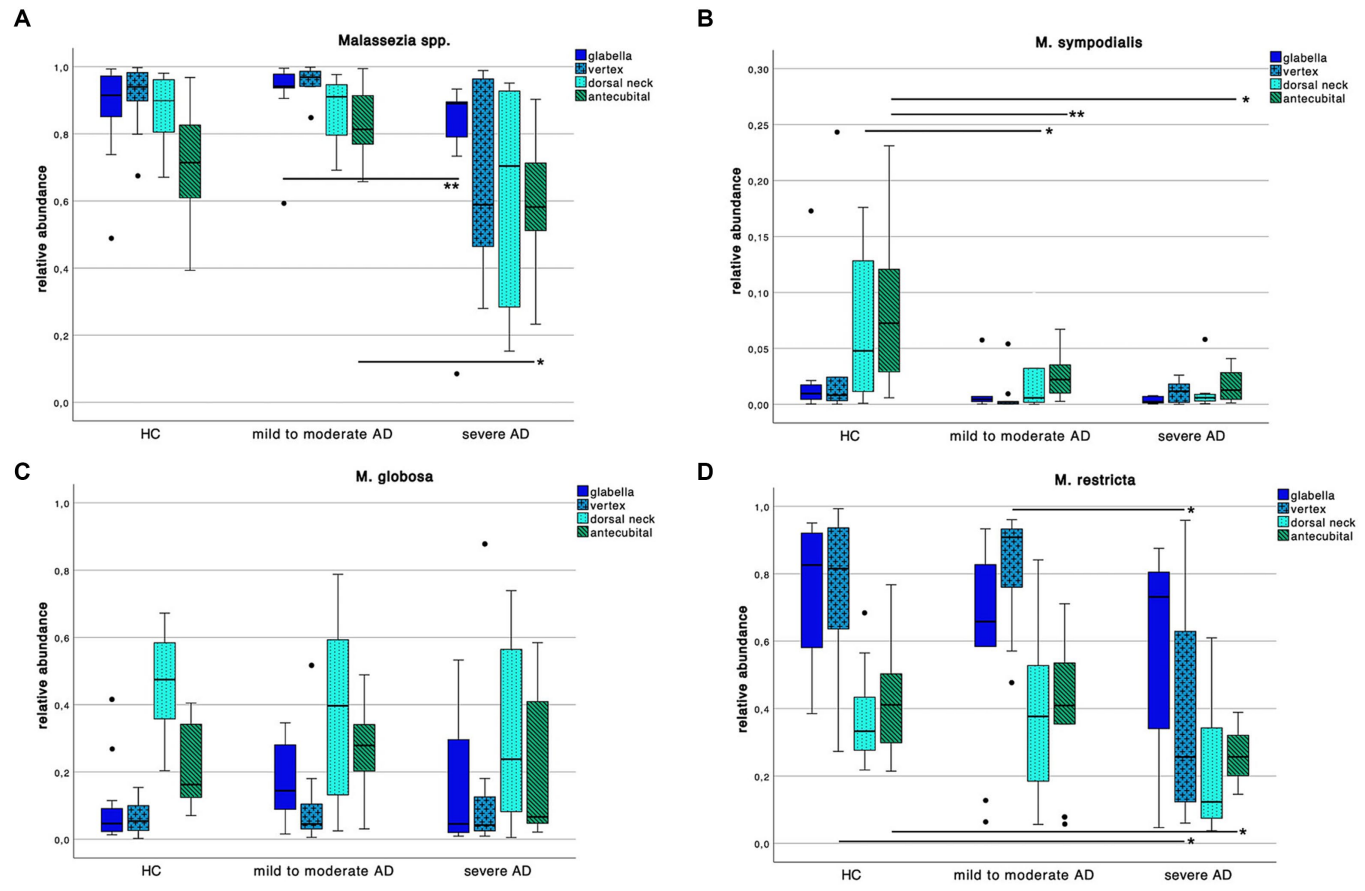
Colonization pattern of *Malassezia* spp. for each disease severity subgroup. Whiskers box plot displaying the relative abundance of **(A)** *Malassezia* spp., **(B)** *M. sympodialis*, **(C)** *M. globosa* and **(D)** *M. restricta* at different skin site (glabella, vertex, dorsal neck, antecubital crease) for every severity subgroup. Pairwise comparison for differences among the colonization pattern were performed using Mann–Whitney-*U* test, whereas * and ** indicate a *p*-value ≤0.05 and ≤0.01, respectively.

We further explored differences in the skin mycobiota abundance of *Malassezia* spp. *M. sympodialis*, *M. restricta*, *M. globosa* as displayed in Figure 5.

We found an overall decrease of *Malassezia* spp. abundance in severe AD compared to HC and mild to moderate AD. Yet only between mild to moderate AD to severe AD at skin site “glabella” and “antecubital” crease we could show a significant decrease (Supplementary Table S2; Mann–Whitney-*U* test, *p* = 0.01 and *p* = 0.03 respectively, Cohen’s effect size *r* = 0.65 and *r* = 0.54 respectively).

Looking at species level, we saw a decrease of *M. sympodialis* abundance in AD compared to HC with significant differences between HC to AD subgroups at antecubital crease swab site (Supplementary Table S2; Mann–Whitney-*U* test, *p* = 0.01 and *p* = 0.02 respectively, Cohen’s effect size *r* = 0.60 and *r* = 0.59 respectively) as well as in HC to mild to moderate AD at dorsal neck swab site (Supplementary Table S2; Mann–Whitney-*U* test, *p* = 0.04, Cohen’s effect size *r* = 0.44). Likewise in *M. restricta* colonization there was a decrease of abundance in severe AD compared to HC and mild to moderate AD at vertex swab site (Supplementary Table S2; Mann–Whitney-*U* test, *p* = 0.04, Cohen’s effect size *r* = 0.44 and *r* = 0.52 respectively) and in severe compared to HC at antecubital crease swab site (Supplementary Table S2; Mann–Whitney-*U* test, *p* = 0.02, Cohen’s effect size *r* = 0.52). There was no difference in *M. globosa* colonization pattern among the 3 different groups.

### Increase in *Candida* spp. colonization in severe AD

**Figure 6:**
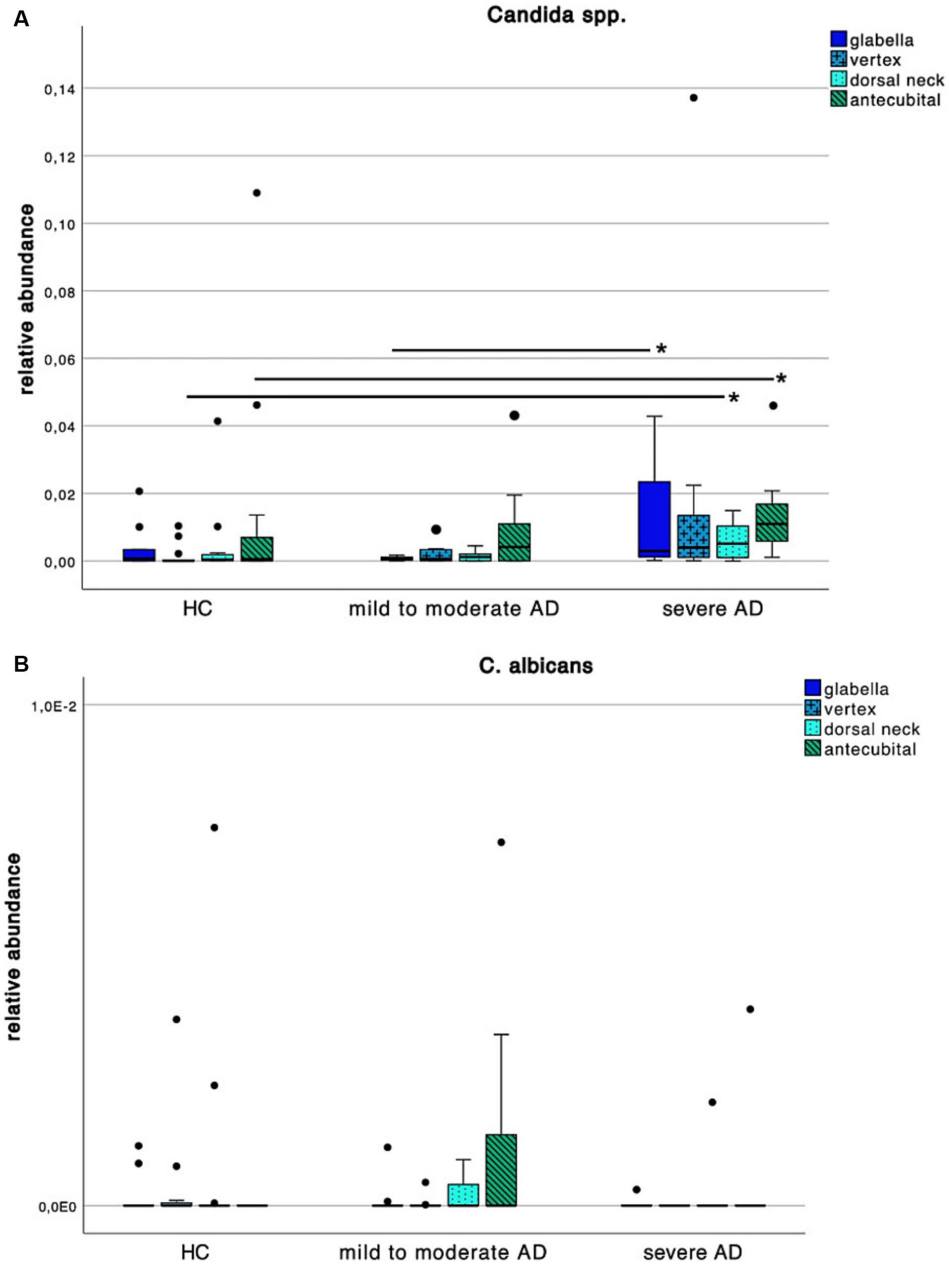
Colonization pattern of *Candida* spp. for each disease severity subgroup. Whiskers box plot displaying the relative abundance of **(A)** *Candida* spp. and **(B)** *C. albicans* at different skin site (glabella, vertex, dorsal neck, antecubital crease) for every severity subgroup. Pairwise comparison for differences among the colonization pattern were performed using Mann–Whitney-*U* test, whereas * and ** indicate a *p*-value ≤0.05 and ≤0.01, respectively.

When looking at *Candida* spp. colonization patterns (Figure 6A), we found an increase of relative abundance in severe AD when compared to HC as well as mild to moderate AD. At the vertex and dorsal neck, this difference was significant between severe AD and HC as well as mild to moderate AD (Supplementary Table S2; Mann–Whitney-*U* test, *p* = 0.02 and *p* = 0.05 respectively, Cohen’s effect size *r* = 0.53 and *r* = 0.43 respectively). At the glabella there was a significant difference in relative abundance between severe AD and mild to moderate AD (Supplementary Table S2; Mann–Whitney-*U* test, *p* = 0.04, Cohen’s effect size *r* = 0.52), for *C. albicans*, we saw a very low relative abundance of fungal colonization on species level with values ranging between 0–0.72% and no detectable difference among the 3 subgroups (Figure 6B).

### Negative correlation between specific sensitization against *Malassezia* spp. and relative abundance of *Malassezia sympodialis*

By assessing the colonization patterns, Spearman’s rank correlation analysis showed a statistically significant, negative correlation between *M. sympodialis* colonization and m227 as well as Mala s 6, respectively, (Supplementary Figures S6A–C). For *Malassezia* spp., *M. restricta* and *M. globosa* colonization, there was a negative yet not significant correlation towards *Malassezia* spp. sensitization.

### Positive correlation between specific sensitization against *Candida albicans* and relative abundance of *Candida* spp.

Correlation analysis for *Candida* spp. and *C. albicans* colonization showed only a significant positive correlation between *Candida* spp. at skin site vertex and *C. albicans* antigen m5 (Supplementary Figure S6D). For other skin sites, correlations were positive yet not significant. For *C. albicans* colonization, there was no correlation with its antigen m5.

## Discussion

### Increase in sensitization, decrease in *Malassezia* spp. colonization

The aim of this study was to examine differences in sensitization towards *Malassezia* spp. and *C. albicans* between AD patients HC and put it in perspective to the skin mycobiota and impact on disease severity. This study shows an inverse correlation between sensitization towards and colonization with *Malassezia* spp. with increasing disease severity in AD patients.

Relative abundance of *Malassezia* spp. in severe AD patients was significantly decreased (Figure 5A) when compared to mild to moderate AD patients. Further, relative abundance of *M. sympodialis* and *M. restricta* decreased significantly with increasing disease severity. Yet sensitization towards *M. sympodialis* (Mala s 6 in ALEX) and the mixture of components of *M. restricta*, *M. globosa* and *M. sympodialis* (m227 in ImmunoCAP) correlated positively with disease severity (Figure 1). When comparing sensitization and colonization, we found a negative correlation between *Malassezia* spp. as well as on species level (*M. sympodialis*, *M. restricta*, *M. globosa*) with only significant results in *M. sympodialis*.

The question arises, how the shown decrease in skin colonization with *Malassezia* spp. can be put into perspective with increasing sensitization. The overall increase of total and specific sensitization with disease severity (Supplementary Table S2; Figures 1–4) with higher levels in AD patients is already well-known (11, 23, 32, 33) and might be attributable to the fact, that our AD population is on the extrinsic end of the phenotypical AD spectrum and thus is prone to react towards common allergens with the production of IgE (2) (Supplementary Figures S2, S3). On the other hand, among all *Malassezia* spp., with exception of *M. pachydermatis*, lipid-dependence for growth is a common feature (8). Pathological features of AD patients next to the increased susceptibility towards allergens and the production of sIgE (9) are the elevation in stratum corneum pH which enhances the activity of degradatory proteases and decreases the activity of lipid synthesis enzymes (6), thus withdrawing the substrate for growth of *Malassezia* spp. (51, 52). Those findings show the potential use of multiplex-assays such as ALEX^2®^ to detect multiple specific IgE to *Malassezia* species (amongst other allergens), making it a valuable diagnostic tool in the management of AD. Results could have a major impact on add on antifungal therapies, being another step to precision medicine.

Considering skin colonization, our findings are consistent with the results from Sandström Falk et al. (53) while other studies have described no difference in skin colonization with *Malassezia* spp. at all (25), or even an increased colonization with *Malassezia* spp. with increasing disease severity in AD patients (26). When comparing these study populations with ours, the one from Sandström et al. is also Caucasian whilst the other two studies have been conducted on an Asian population. There is evidence, that the skin mycobiota differs among different ethnicities (8, 21, 54) which might be attributed not only to geographical differences but also to different sampling techniques (53). There has even been a description of different specific sensitization towards *Malassezia* spp. in a sub-Saharan AD population whilst no difference in skin colonization was found (55). Thus, we assume that with increase in inflammation and withdrawal of lipids as a substrate, colonization with *Malassezia* spp. decreases. Further, we conclude that ethnicity has an impact on the skin mycobiota which makes it difficult to draw conclusions from our study population when compared to other ethnicities. Our findings underline, that ethnic and geographical differences among AD patients could have an impact on a therapeutic level. A distinct group could benefit from antifungal treatment, which could be easily available in wide regions off the world (compared to other systemic therapies) (3).

### Increase in *Candida* spp. colonization

Interestingly along the reduced frequency in skin colonization with *Malassezia* spp. we saw an increase with *Candida* spp. in severe AD patients (Figure 6A).

The increase in skin colonization with *Candida* spp. at the expense of *Malassezia* spp. is consistent with other studies (30, 56). It is hypothesized that the skin mycobiota is affected by topical and systemic use of corticosteroids and antibiotics. With more frequent use in severe AD it might be attributable to the change in the composition of skin flora eventually resulting in conditions that favor growth of, i.e., *Candida* spp. (57). When looking at species level, relative abundance of *C. albicans* was very low with maximum values under 1% and with no difference among HC and AD. Looking at our study population, there is no relevant difference in intake or application of topical or systemic antibiotics or corticosteroids, respectively. Further, only two patients used topical antimycotics, which did not show a relevant effect on colonization. Another hypothesis is, that *Candida* spp., i.e., being one of the most common pathogenic yeasts of normal skin flora (29) could either profit from or generate an inflammatory environment leading to the above mentioned decrease in lipid synthesis (6) and thus decrease in colonization with *Malassezia* spp. A recent meta-analysis from He et al. (27) has shown a superiority in treatment of AD using topical antifungals compared to topical glucocorticoids. This suggests that the mycobiota is of central cause in AD progression (17) and already established topical antifungal therapy, nowadays consisting mostly of azole derivates (17, 27, 58), could reduce the amount of allergens causing a type I inflammatory response (35, 59).

Along with the increase in skin colonization the severe AD patients showed significant sensitization towards *C. albicans* which is in line with the above-mentioned increased specific sensitization in our polysensitized severe AD population and is consistent with findings of other studies (28).

Further investigation concerning causality between inflammatory processes, fungal skin colonization and sensitization towards skin mycobiota could have a major impact on targeted and maybe even preventive management in AD patients. With this assumption, our study could also lay a headstone for clinical investigative trials to recess antifungal treatment approaches in bigger cohorts. This was beyond the scope of our study and with our AD study population counting only 16 patients, and only two (13%) of them using topical antifungals, it would have been difficult to draw conclusion, making sample size one of the major limitations of our study.

### Limitations

The sensitization pattern was primarily assessed using the relatively newly developed ALEX^2®^, a macroarray that allows to obtain multiplex sIgE profile as well as tIgE (60, 61). Due to the removal of *C. albicans* extract in ALEX^2®^ compared to the ALEX^1®^, we could not compare the sensitization pattern using this multiplex assay only. Further, ALEX^2®^ could only measure sensitization towards *M. sympodialis*, while lacking all the other *Malassezia* spp., especially *M. restricta* and *M. globosa*, who are the most abundant on human skin (62, 63), making it one of the major drawbacks of this study. To expand the specific sensitization measurement towards *C. albicans*, *M. restricta* and *M. globosa*, we had to use the ImmunoCAP^®^ assay.

To reduce the influence of fungal colonization of non-measured skin sites on systemic sensitization, we selected lipid rich skin swab sites where we expected *Malassezia* spp. to be present in a high number. Nevertheless, the possibility that the sIgE towards *Malassezia* spp. is elevated due to colonization of other skin sites and colonization in total is underestimated with increasing disease severity cannot be excluded.

Another already above-mentioned limitation is the small sample size which makes our study population vulnerable to confounders such as sex, topical and systemic medication, and skin care regimens. Further, we saw differences especially concerning colonization patterns among HC and AD patients, with many of them not being significant. This might as well be due to the very small sample size.

### Strengths

One of the strengths of this study is the direct comparison of specific sensitization towards the allergen and the allergen load, i.e., sIgE of *Malassezia* spp. and *C. albicans* with and their abundance on human skin. This is the first study using this design on a Caucasian study population while comparing AD with HC with only one study from Zhang et al. (25) using a similar approach on AD patients only. With assessing tIgE and sIgE using ImmunoCAP^®^, we were able to use the best studied sIgE single-and multiplex assay (64). It shows substantial to good accordance to ALEX^®^ (61), and we were able to cross-validate the total and specific sensitization which correlated strongly (Supplementary Figure S4).

A further strength lies in the use of a PCR-approach to measure fungal skin colonization. *Malassezia* spp. are very difficult to cultivate and even if growth occurs, some species grow much slower than others and thus would be overwhelmed, leading a wrong representation of *Malassezia* spp. which has been described as cultivation bias (62).

## Conclusion/outlook

With this study we gathered further evidence for increased specific sensitization as well as change in composition of skin mycobiota in severe AD patients. Further investigation with larger sample size to put these findings into a clinical context need to be done.

## Data availability statement

The datasets presented in this study can be found in online repositories. The names of the repository/repositories and accession number(s) can be found at: https://www.ebi.ac.uk/ena/browser/view/PRJEB44167.

## Ethics statement

The studies involving humans were approved by Kantonale Ethikkommission Zürich; EK 2016-00301. The studies were conducted in accordance with the local legislation and institutional requirements. The participants provided their written informed consent to participate in this study.

## Author contributions

LS: Writing – original draft, Writing – review & editing, Conceptualization, Investigation, Methodology, Project administration, Visualization, Formal analysis, Software. BS: Conceptualization, Investigation, Methodology, Project administration, Writing – original draft, Writing – review & editing, Formal analysis, Software. PB: Conceptualization, Supervision, Writing – review & editing. PS-G: Writing – review & editing, Conceptualization, Supervision, Funding acquisition, Project administration, Resources, Validation. M-CB: Funding acquisition, Project administration, Resources, Supervision, Validation, Writing – review & editing, Conceptualization. CL: Funding acquisition, Project administration, Resources, Supervision, Validation, Writing – review & editing, Conceptualization, Methodology.
