# Supplementary material for: Decreased skin colonization with Malassezia spp. and increased skin colonization with Candida spp. in patients with severe atopic dermatitis
Source: Front Med (Lausanne). 2024 Feb 20;11:1353784. doi: 10.3389/fmed.2024.1353784 (PMC10912638; doi:10.3389/fmed.2024.1353784)
Supplement: Supplementary file 1 [file Data_Sheet_1.docx]

**Supplementary Material**

**Supplementary Tables**

**Supplementary Table S1: ALEX^2®^** **allergen list (**according to company information: https://www.macroarraydx.com)[Accessed March 25, 2023].

| **Allergen-ID** | **Allergen-code** | **Common name** | **Scientific name** | **Component** | **Biochemical designation** | **Component (C) / Extract (E)** |
| --- | --- | --- | --- | --- | --- | --- |
| 1 | NA | tIgE | tIgE | tIgE | tIgE | tIgE |
| 3 | t19 | Acacia | Acacia mimosa |  |  | E |
| 4 | d70 | Acarus siro | Acarus siro |  |  | E |
| 5 | f526 | House cricket | Acheta domesticus |  |  | E |
| 6 | f500 | Kiwi | Actinidia deliciosa | nAct d 1 | Cysteine Protease | C |
| 7 | f502 | Kiwi | Actinidia deliciosa | nAct d 10 | nsLTP | C |
| 8 | f503 | Kiwi | Actinidia deliciosa | nAct d 2 | TLP | C |
| 9 | f501 | Kiwi | Actinidia deliciosa | nAct d 5 | Kiwellin | C |
| 10 | t304 | Tree of heaven | Ailanthus altissima |  |  | E |
| 14 | f48 | Onion | Allium cepa |  |  | E |
| 15 | f47 | Garlic | Allium sativum |  |  | E |
| 16 | t100 | Alder | Alnus glutinosa | rAln g 1 | PR-10 | C |
| 17 | t101 | Alder | Alnus glutinosa | rAln g 4 | Polcalcin | C |
| 18 | m229 | Alternaria alternata | Alternaria alternata | rAlt a 1 | Alt a 1-Family | C |
| 19 | m230 | Alternaria alternata | Alternaria alternata | rAlt a 6 | Enolase | C |
| 20 | w14 | Pigweed | Amaranthus retroflexus |  |  | E |
| 21 | w1 | Ragweed | Ambrosia artemisiifolia |  |  | E |
| 22 | w230 | Ragweed | Ambrosia artemisiifolia | rAmb a 1 | Pectate Lyase | C |
| 23 | w300 | Hemp (CBD) | Cannabis sativa |  |  | E |
| 24 | f202 | Cashew | Anacardium occidentale |  |  | E |
| 25 | f550 | Cashew | Anacardium occidentale | rAna o 2 | 11S Globulin | C |
| 26 | f443 | Cashew | Anacardium occidentale | rAna o 3 | 2S Albumin | C |
| 27 | p10 | Anisakis simplex | Anisakis simplex | rAni s 1 | Kunitz Serin Protease Inhibitor | C |
| 28 | p11 | Anisakis simplex | Anisakis simplex | rAni s 3 | Tropomyosin | C |
| 29 | f417 | Celery | Apium graveolens | rApi g 1 | PR-10 | C |
| 30 | f504 | Celery | Apium graveolens | rApi g 2 | nsLTP | C |
| 31 | f505 | Celery | Apium graveolens | rApi g 6 | nsLTP | C |
| 32 | i1 | Honey bee venom | Apis mellifera |  |  | E |
| 33 | i208 | Honey bee venom | Apis mellifera | nApi m 1 | Phospholipase A2 | C |
| 34 | i217 | Honey bee venom | Apis mellifera | rApi m 10 | Icarapin Variant 2 | C |
| 35 | f422 | Peanut | Arachis hypogea | nAra h 1 | 7/8S Globulin | C |
| 36 | f803 | Peanut | Arachis hypogea | rAra h 15 | Oleosin | C |
| 37 | f423 | Peanut | Arachis hypogea | rAra h 2 | 2S Albumin | C |
| 38 | f424 | Peanut | Arachis hypogea | nAra h 3 | 11S Globulin | C |
| 39 | f447 | Peanut | Arachis hypogea | rAra h 6 | 2S Albumin | C |
| 40 | f352 | Peanut | Arachis hypogea | rAra h 8 | PR-10 | C |
| 41 | f427 | Peanut | Arachis hypogea | rAra h 9 | nsLTP | C |
| 42 | o100 | Pigeon tick | Argas reflexus | rArg r 1 | Lipocalin | C |
| 43 | w6 | Mugwort | Artemisia vulgaris |  |  | E |
| 44 | w231 | Mugwort | Artemisia vulgaris | rArt v 1.0101 | Plant Defensin | C |
| 45 | w233 | Mugwort | Artemisia vulgaris | rArt v 3.0201 | nsLTP | C |
| 46 | m218 | Aspergillus fumigatus | Aspergillus fumigatus | rAsp f 1 | Mitogillin Family | C |
| 47 | m220 | Aspergillus fumigatus | Aspergillus fumigatus | rAsp f 3 | Peroxysomal Protein | C |
| 48 | m221 | Aspergillus fumigatus | Aspergillus fumigatus | rAsp f 4 | unknown | C |
| 49 | m222 | Aspergillus fumigatus | Aspergillus fumigatus | rAsp f 6 | Mn Superoxid-Dismutase | C |
| 50 | f7 | Oat | Avena sativa |  |  | E |
| 51 | f18 | Brazil nut | Bertholletia excelsa |  |  | E |
| 52 | f354 | Brazil nut | Bertholletia excelsa | rBer e 1 | 2S Albumin | C |
| 53 | t215 | Silver birch | Betula verrucosa | rBet v 1 | PR-10 | C |
| 54 | t216 | Silver birch | Betula verrucosa | rBet v 2 | Profilin | C |
| 55 | t225 | Silver birch | Betula verrucosa | rBet v 6 | Isoflavon Reductase | C |
| 56 | i100 | German cockroach | Blatella germanica | rBla g 1 | Cockroach group 1 | C |
| 57 | i101 | German cockroach | Blatella germanica | rBla g 2 | Aspartyl protease | C |
| 58 | i102 | German cockroach | Blatella germanica | rBla g 4 | Calycin | C |
| 59 | i103 | German cockroach | Blatella germanica | rBla g 5 | Glutathione S-transferase | C |
| 60 | i301 | German cockroach | Blatella germanica | rBla g 9 | Arginine Kinase | C |
| 61 | d301 | Blomia tropicalis | Blomia tropicalis | rBlo t 10 | Tropomyosin | C |
| 62 | d302 | Blomia tropicalis | Blomia tropicalis | rBlo t 21 | unknown | C |
| 63 | d300 | Blomia tropicalis | Blomia tropicalis | rBlo t 5 | Mite, Group 5 | C |
| 64 | e100 | Cattle | Bos domesticus | rBos d 2 | Lipocalin | C |
| 65 | f76 | Cow's milk | Bos domesticus | nBos d 4 | α-Lactalbumin | C |
| 66 | f77 | Cow's milk | Bos domesticus | nBos d 5 | β-Lactoglobulin | C |
| 67 | e204 | Beef | Bos domesticus | nBos d 6 | Serum Albumin | C |
| 68 | f78 | Cow's milk | Bos domesticus | nBos d 8 | Casein | C |
| 69 | f27 | Beef | Bos domesticus |  |  | E |
| 70 | f2 | Cow's milk | Bos domesticus |  |  | E |
| 71 | t305 | Paper mulberry | Broussonetia papyrifera |  |  | E |
| 72 | f506 | Camel's milk | Camelus dromedarius |  |  | E |
| 73 | e101 | Dog | Canis familiaris | rCan f 1 | Lipocalin | C |
| 74 | e102 | Dog | Canis familiaris | rCan f 2 | Lipocalin | C |
| 75 | e221 | Dog | Canis familiaris | nCan f 3 | Serum Albumin | C |
| 76 | e302 | Dog | Canis familiaris | rCan f 4 | Lipocalin | C |
| 77 | e303 | Dog | Canis familiaris | rCan f 6 | Lipocalin | C |
| 78 | e304 | Dog | Canis familiaris | rCan f Fel d 1 like | Fel d 1 like | C |
| 79 | e308 | Dog, male urine (incl. Can f 5) | Canis familiaris |  |  | E |
| 80 | w302 | Hemp | Cannabis sativa | rCan s 3 | nsLTP | C |
| 81 | w301 | Annual mercury | Mercurialis annua | rMer a 1 | Profilin | C |
| 82 | f218 | Paprika | Capsicum annuum |  |  | E |
| 83 | e80 | Goat | Capra hircus |  |  | E |
| 84 | f300 | Goat's milk | Capra hircus |  |  | E |
| 85 | f265 | Caraway | Carum carvi |  |  | E |
| 86 | f201 | Pecan | Carya illinoensis |  |  | E |
| 87 | f293 | Papaya | Carica papaya |  |  | E |
| 88 | e305 | Guinea pig | Cavia porcellus | rCav p 1 | Lipocalin | C |
| 89 | w10 | Lamb's quarter | Chenopodium album |  |  | E |
| 90 | w100 | Lamb's quarter | Chenopodium album | rChe a 1 | Ole e 1-Family | C |
| 91 | f347 | Quinoa | Chenopodium quinoa |  |  | E |
| 92 | f23 | Crab | Chionoecetes spp. |  |  | E |
| 93 | f309 | Chickpea | Cicer arietinus |  |  | E |
| 94 | f33 | Orange | Citrus sinensis |  |  | E |
| 95 | m2 | Cladosporium herbarum | Cladosporium herbarum |  |  | E |
| 96 | m100 | Cladosporium herbarum | Cladosporium herbarum | rCla h 8 | Short Chain Dehydrogenase | C |
| 97 | f205 | Atlantic herring | Clupea harengus |  |  | E |
| 98 | f525 | Atlantic herring | Clupea harengus | rClu h 1 | β-Parvalbumin | C |
| 99 | t102 | Hazel | Corylus avellana | rCor a 1.0103 | PR-10 | C |
| 100 | f428 | Hazelnut | Corylus avellana | rCor a 1.0401 | PR-10 | C |
| 101 | f522 | Hazelnut | Corylus avellana | nCor a 11 | 7/8S Globulin | C |
| 102 | RUO-f800 | Hazelnut | Corylus avellana | rCor a 12 | Oleosin | C |
| 103 | f439 | Hazelnut | Corylus avellana | rCor a 14 | 2S Albumin | C |
| 104 | f425 | Hazelnut | Corylus avellana | rCor a 8 | nsLTP | C |
| 105 | f440 | Hazelnut | Corylus avellana | nCor a 9 | 11S Globulin | C |
| 106 | t4 | Hazel | Corylus avellana |  |  | E |
| 107 | f529 | Brown shrimp | Crangon crangon | rCra c 6 | Troponin C | C |
| 108 | t303 | Sugi | Cryptomeria japonica | rCry j 1 | Pectate Lyase | C |
| 109 | f528 | Muskmelon | Cucumis melo | rCuc m 2 | Profilin | C |
| 110 | f226 | Pumpkin seed | Cucurbita pepo |  |  | E |
| 111 | t226 | Arizona cypress | Cupressus arizonica | nCup a 1 | Pectate Lyase | C |
| 112 | t222 | Cypress | Cupressus sempervirens |  |  | E |
| 113 | g2 | Bermuda grass | Cynodon dactylon |  |  | E |
| 114 | g216 | Bermuda grass | Cynodon dactylon | rCyn d 1 | Beta-Expansin | C |
| 115 | f355 | Carp | Cyprinus carpio | rCyp c 1 | β-Parvalbumin | C |
| 116 | f31 | Carott | Daucus carota |  |  | E |
| 117 | f507 | Carott | Daucus carota | rDau c 1 | PR-10 | C |
| 118 | d100 | American house dust mite | Dermatophagoides farinae | rDer f 1 | Cysteine Protease | C |
| 119 | d101 | American house dust mite | Dermatophagoides farinae | rDer f 2 | NPC2 Family | C |
| 120 | d202 | European house dust mite | Dermatophagoides pteronyssinus | rDer p 1 | Cysteine Protease | C |
| 121 | d205 | European house dust mite | Dermatophagoides pteronyssinus | rDer p 10 | Tropomyosin | C |
| 122 | d102 | European house dust mite | Dermatophagoides pteronyssinus | rDer p 11 | Myosin, heavy chain | C |
| 123 | d203 | European house dust mite | Dermatophagoides pteronyssinus | rDer p 2 | NPC2 Family | C |
| 124 | d303 | European house dust mite | Dermatophagoides pteronyssinus | rDer p 20 | Arginine Kinase | C |
| 125 | d304 | European house dust mite | Dermatophagoides pteronyssinus | rDer p 21 | unknown | C |
| 126 | d209 | European house dust mite | Dermatophagoides pteronyssinus | rDer p 23 | Peritrophin-like protein domain | C |
| 127 | d103 | European house dust mite | Dermatophagoides pteronyssinus | rDer p 5 | unknown | C |
| 128 | d104 | European house dust mite | Dermatophagoides pteronyssinus | rDer p 7 | Mite Group 7 | C |
| 129 | i25 | Long-headed wasp venom | Dolichovespula spp. |  |  | E |
| 130 | e227 | Horse | Equus caballus | rEqu c 1 | Lipocalin | C |
| 131 | e306 | Horse | Equus caballus | nEqu c 3 | Serum Albumin | C |
| 132 | e307 | Horse | Equus caballus | rEqu c 4 | Latherin | C |
| 133 | f321 | Horse | Equus caballus |  |  | E |
| 134 | f286 | Mare's milk | Equus caballus |  |  | E |
| 135 | f11 | Buckwheat | Fagopyrum esculentum |  |  | E |
| 136 | f508 | Buckwheat | Fagopyrum esculentum | nFag e 2 | 2S Albumin | C |
| 137 | t300 | Beech | Fagus sylvatica | rFag s 1 | PR-10 | C |
| 138 | e94 | Cat | Felis domesticus | rFel d 1 | Uteroglobin | C |
| 139 | e220 | Cat | Felis domesticus | nFel d 2 | Serum Albumin | C |
| 140 | e228 | Cat | Felis domesticus | rFel d 4 | Lipocalin | C |
| 141 | e300 | Cat | Felis domesticus | rFel d 7 | Lipocalin | C |
| 142 | k81 | Weeping fig | Ficus benjamina |  |  | E |
| 143 | f328 | Fig | Ficus carica |  |  | E |
| 144 | f44 | Strawberry | Fragaria ananassa | rFra a 1 + rFra a 3 | PR-10+LTP | C-Mix |
| 145 | t25 | Ash | Fraxinus excelsior |  |  | E |
| 146 | t103 | Ash | Fraxinus excelsior | rFra e 1 | Ole e 1-Family | C |
| 147 | f3 | Atlantic cod | Gadus morhua |  |  | E |
| 148 | f509 | Atlantic cod | Gadus morhua | nGad m 1 | β-Parvalbumin | C |
| 149 | f805 | Atlantic cod | Gadus morhua | nGad m 2&3 | β-Enolase&Aldolase | C-Mix |
| 150 | f233 | Egg white | Gallus domesticus | nGal d 1 | Ovomucoid | C |
| 151 | f232 | Egg white | Gallus domesticus | nGal d 2 | Ovalbumin | C |
| 152 | f323 | Egg white | Gallus domesticus | nGal d 3 | Ovotransferrin | C |
| 153 | k208 | Egg white | Gallus domesticus | nGal d 4 | Lysozym C | C |
| 154 | f510 | Egg yolk | Gallus domesticus | nGal d 5 | Serum Albumin | C |
| 155 | f83 | Chicken | Gallus domesticus |  |  | E |
| 156 | f1 | Egg white | Gallus domesticus |  |  | E |
| 157 | f75 | Egg yolk | Gallus domesticus |  |  | E |
| 158 | d105 | Glycyphagus domesticus | Glycyphagus domesticus | rGly d 2 | NPC2 Family | C |
| 159 | f353 | Soy | Glycine max | rGly m 4 | PR-10 | C |
| 160 | f431 | Soy | Glycine max | rGly m 5 | 7/8S Globulin | C |
| 161 | f432 | Soy | Glycine max | nGly m 6 | 11S Globulin | C |
| 162 | f511 | Soy | Glycine max | nGly m 8 | 2S Albumin | C |
| 163 | k84 | Sunflower seed | Helianthus annuus |  |  | E |
| 164 | k215 | Latex | Hevea brasiliensis | rHev b 1 | Rubber elongation factor | C |
| 165 | k224 | Latex | Hevea brasiliensis | rHev b 11 | Class 1 Chitinase | C |
| 166 | k217 | Latex | Hevea brasiliensis | rHev b 3 | small rubber particle protein | C |
| 167 | k218 | Latex | Hevea brasiliensis | rHev b 5 | unknown | C |
| 168 | k220 | Latex | Hevea brasiliensis | rHev b 6.02 | Pro-Hevein | C |
| 169 | k221 | Latex | Hevea brasiliensis | rHev b 8 | Profilin | C |
| 170 | f80 | Lobster | Homarus gammarus |  |  | E |
| 171 | o214 | Hom s lactoferrin |  | rHom s LF | CCD | C |
| 172 | f6 | Barley | Hordeum vulgare |  |  | E |
| 173 | f441 | Walnut | Juglans regia | nJug r 1 | 2S Albumin | C |
| 174 | f512 | Walnut | Juglans regia | nJug r 2 | 7/8S Globulin | C |
| 175 | f539 | Walnut | Juglans regia | rJug r 3 | nsLTP | C |
| 176 | f540 | Walnut | Juglans regia | nJug r 4 | 11S Globulin | C |
| 177 | f541 | Walnut | Juglans regia | nJug r 6 | 7/8S Globulin | C |
| 178 | t10 | Walnut | Juglans regia |  |  | E |
| 179 | t63 | Mountain cedar | Juniperus ashei |  |  | E |
| 180 | f235 | Lentil | Lens culinaris |  |  | E |
| 181 | d305 | Lepidoglyphus destructor | Lepidoglyphus destructor | rLep d 2 | NPC2 Family | C |
| 182 | f24 | Shrimp mix | Litopaenaeus setiferus, Farfantepenaeus aztecus, Farfantepenaeus dourarum |  |  | E |
| 183 | f553 | Migratory locust | Locusta migratoria |  |  | E |
| 184 | g100 | Perennial ryegrass | Lolium perenne | rLol p 1 | Beta-Expansin | C |
| 185 | f258 | Squid | Loligo spp. |  |  | E |
| 186 | f335 | Lupine seed | Lupinus albus |  |  | E |
| 187 | f513 | Macadamia | Macadamia integrifolia | nMac i 2S Albumin | 2S Albumin | C |
| 188 | f345 | Macadamia | Macadamia integrifolia |  |  | E |
| 189 | f434 | Apple | Malus domestica | rMal d 1 | PR-10 | C |
| 190 | f514 | Apple | Malus domestica | nMal d 2 | TLP | C |
| 191 | f435 | Apple | Malus domestica | rMal d 3 | nsLTP | C |
| 192 | y5 | Malassezia sympodialis | Malassezia sympodialis | rMala s 11 | Mn Superoxid-Dismutase | C |
| 193 | y2 | Malassezia sympodialis | Malassezia sympodialis | rMala s 5 | unknown | C |
| 194 | y3 | Malassezia sympodialis | Malassezia sympodialis | rMala s 6 | Cyclophilin | C |
| 195 | f91 | Mango | Mangifera indica |  |  | E |
| 196 | f284 | Turkey | Meleagris gallopavo |  |  | E |
| 197 | w300 | Ragweed | Ambrosia artemisiifolia | rAmb a 4 | Plant Defensin | C |
| 198 | RUO-e800 | Golden hamster | Mesocricetus auratus | rMes a 1 | Lipocalin | C |
| 199 | t71 | Mulberry tree | Morus rubra |  |  | E |
| 200 | f92 | Banana | Musa acuminata |  |  | E |
| 201 | e103 | Mouse | Mus musculus | rMus m 1 | Lipocalin | C |
| 202 | f37 | Common mussel | Mytilus edulis |  |  | E |
| 203 | t224 | Olive | Olea Europaea | nOle e 1 | Ole e 1-Family | C |
| 204 | RUO-t227 | Olive | Olea Europaea | rOle e 7 | nsLTP | C |
| 205 | t240 | Olive | Olea Europaea | rOle e 9 | 1,3 β Glucanase | C |
| 206 | f283 | Oregano | Origanum vulgare |  |  | E |
| 207 | e309 | Rabbit | Oryctolagus cuniculus | rOry c 1 | Lipocalin | C |
| 208 | e310 | Rabbit | Oryctolagus cuniculus | rOry c 2 | Lipocalin | C |
| 209 | e311 | Rabbit | Oryctolagus cuniculus | rOry c 3 | Secretoglobin | C |
| 210 | f9 | Rice | Oryza sativa |  |  | E |
| 211 | f213 | Rabbit | Oryctolagus spp. |  |  | E |
| 212 | f290 | Oyster | Ostrea edulis |  |  | E |
| 213 | e81 | Sheep | Ovis aries |  |  | E |
| 214 | f88 | Lamb | Ovis aries |  |  | E |
| 215 | f325 | Sheep's milk | Ovis aries |  |  | E |
| 216 | f515 | Northern prawn | Pandalus borealis |  |  | E |
| 217 | f55 | Millet | Panicum miliaceum |  |  | E |
| 218 | f224 | Poppy seed | Papaver somniferum |  |  | E |
| 219 | f516 | Poppy seed | Papaver somniferum | nPap s 2S Albumin | 2S Albumin | C |
| 220 | w21 | Wall pellitory | Parietaria judaica |  |  | E |
| 221 | w211 | Wall pellitory | Parietaria judaica | rPar j 2 | nsLTP | C |
| 222 | g17 | Bahia grass | Paspalum notatum |  |  | E |
| 223 | f338 | Scallop | Pecten spp. |  |  | E |
| 224 | m1 | Penicilium chrysogenum | Penicilium chrysogenum |  |  | E |
| 225 | f517 | Black-Tiger shrimp | Penaeus monodon | rPen m 1 | Tropomyosin | C |
| 226 | f545 | Black-Tiger shrimp | Penaeus monodon | rPen m 2 | Arginine Kinase | C |
| 227 | f552 | Black-Tiger shrimp | Penaeus monodon | rPen m 3 | Myosin light chain | C |
| 228 | f524 | Black-Tiger shrimp | Penaeus monodon | rPen m 4 | Sarcoplasmic Calcium Binding Protein | C |
| 229 | i206 | American cockroach | Periplaneta americana |  |  | E |
| 230 | i300 | American cockroach | Periplaneta americana | rPer a 7 | Tropomyosin | C |
| 231 | f96 | Avocado | Persea americana |  |  | E |
| 232 | f86 | Parsley | Petroselinum crispum |  |  | E |
| 233 | f315 | Green bean | Phaseolus vulgaris |  |  | E |
| 234 | g205 | Timothy | Phleum pratense | rPhl p 1 | Beta-Expansin | C |
| 235 | g212 | Timothy | Phleum pratense | rPhl p 12 | Profilin | C |
| 236 | g206 | Timothy | Phleum pratense | rPhl p 2 | Expansin | C |
| 237 | g215 | Timothy | Phleum pratense | rPhl p 5.0101 | grass Group 5/6 | C |
| 238 | g209 | Timothy | Phleum pratense | rPhl p 6 | grass Group 5/6 | C |
| 239 | g210 | Timothy | Phleum pratense | rPhl p 7 | Polcalcin | C |
| 240 | t105 | Date palm | Phoenix dactylifera | rPho d 2 | Profilin | C |
| 241 | e301 | Djungarian hamster | Phodopus sungorus | rPhod s 1 | Lipocalin | C |
| 242 | g7 | Common reed | Phragmites communis |  |  | E |
| 243 | f271 | Anise | Pimpinella anisum |  |  | E |
| 244 | f12 | Pea | Pisum sativum |  |  | E |
| 245 | f531 | Pistachio | Pistacia vera | rPis v 1 | 2S Albumin | C |
| 246 | f532 | Pistachio | Pistacia vera | nPis v 2 | 11S Globulin subunit | C |
| 247 | f533 | Pistachio | Pistacia vera | nPis v 3 | 7/8S Globulin | C |
| 248 | RUO-f804 | Pistachio | Pistacia vera | rPis v 4 | Mn Superoxid-Dismutase | C |
| 249 | t241 | London plane tree | Platanus acerifolia | rPla a 1 | Plant Invertase | C |
| 250 | t301 | London plane tree | Platanus acerifolia | nPla a 2 | Polygalacturonase | C |
| 251 | t302 | London plane tree | Platanus acerifolia | rPla a 3 | nsLTP | C |
| 252 | w9 | Ribwort | Plantago lanceolata |  |  | E |
| 253 | w234 | Ribwort | Plantago lanceolata | rPla l 1 | Ole e 1-Family | C |
| 254 | i4 | Paper wasp venom | |  |  | E |
| 255 | i210 | Paper wasp venom | Polistes dominulus | rPol d 5 | Antigen 5 | C |
| 256 | t14 | Cottonwood | Populus nigra |  |  | E |
| 257 | f242 | Cherry | Prunus spp. |  |  | E |
| 258 | f20 | Almond | Prunus dulcis |  |  | E |
| 259 | f420 | Peach | Prunus persica | Pru p 3 | nsLTP | C |
| 260 | RUO-f801 | Peach | Prunus persica | rPru p 7 | Gibberellin-regulated protein | C |
| 261 | f94 | Pear | Pyrus communis |  |  | E |
| 262 | f535 | Thornback ray | Raja clavata |  |  | E |
| 263 | f536 | Thornback ray | Raja clavata | rRaj c Parvalbumin | α-Parvalbumin | C |
| 264 | e73 | Rat | Rattus norvegicus |  |  | E |
| 265 | f207 | Venus clam | Ruditapes spp. |  |  | E |
| 266 | f45 | Baker's yeast | Sacccaromyces cerevisiae |  |  | E |
| 267 | w11 | Russian thistle | Salsola kali |  |  | E |
| 268 | w232 | Russian thistle | Salsola kali | rSal k 1 | Pectin Methylesterase | C |
| 269 | f41 | Salmon | Salmo salar |  |  | E |
| 270 | f534 | Salmon | Salmo salar | rSal s 1 | β-Parvalbumin | C |
| 271 | f206 | Atlantic mackerel | Scomber scombrus |  |  | E |
| 272 | f551 | Atlantic mackerel | Scomber scombrus | rSco s 1 | β-Parvalbumin | C |
| 273 | f5 | Cultivated rye | Secale cereale |  |  | E |
| 274 | g12 | Rye | Secale cereale |  |  | E |
| 275 | f10 | Sesame | Sesamum indicum |  |  | E |
| 276 | f518 | Sesame | Sesamum indicum | nSes i 1 | 2S Albumin | C |
| 277 | f89 | Mustard | Brassica / Sinapis spp. |  |  | E |
| 278 | f519 | Mustard | Brassica / Sinapis spp. | rSin a 1 | 2S Albumin | C |
| 279 | i70 | Fire ant venom | Solenopsis richteri & Solenopsis invicta |  |  | E |
| 280 | f35 | Potato | Solanum tuberosum |  |  | E |
| 281 | f25 | Tomato | Solanum lycopersicum |  |  | E |
| 282 | f520 | Tomato | Solanum lycopersicum | rSola l 6 | nsLTP | C |
| 283 | f530 | Pig | Sus domesticus | rSus d 1 | Serum Albumin | C |
| 284 | e83 | Pig | Sus domesticus |  |  | E |
| 285 | f26 | Pig | Sus domesticus |  |  | E |
| 286 | f527 | Mealworm | Tenebrio molitor |  |  | E |
| 287 | f40 | Tuna | Thunnus albacares |  |  | E |
| 288 | f538 | Tuna | Thunnus albacares | Thu a 1 | β-Parvalbumin | C |
| 289 | f542 | Wheat | Triticum aestivum | rTri a 14 | nsLTP | C |
| 290 | f543 | Wheat | Triticum aestivum | rTri a 19 | Omega-5-Gliadin | C |
| 291 | f544 | Wheat | Triticum aestivum | nTri a aA_TI | Alpha-Amylase Trypsin-Inhibitor | C |
| 292 | f305 | Fenugreek seeds | Trigonella foenum-graecum |  |  | E |
| 293 | f124 | Spelt | Triticum spelta |  |  | E |
| 294 | d72 | Tyrophagus putrescentiae | Tyrophagus putrescentiae |  |  | E |
| 295 | d306 | Tyrophagus putrescentiae | Tyrophagus putrescentiae | rTyr p 2 | NPC2 Family | C |
| 296 | t8 | Elm | Ulmus campestris |  |  | E |
| 297 | w20 | Nettle | Urtica dioica |  |  | E |
| 299 | f288 | Blueberry | Vaccinium myrtillus |  |  | E |
| 300 | i3 | Common wasp venom | Vespula vulgaris |  |  | E |
| 301 | i211 | Common wasp venom | Vespula vulgaris | rVes v 1 | Phospholipase A1 | C |
| 302 | i209 | Common wasp venom | Vespula vulgaris | rVes v 5 | Antigen 5 | C |
| 303 | f521 | Grape | Vitis vinifera | rVit v 1 | nsLTP | C |
| 304 | f537 | Swordfish | Xiphias gladius | rXip g 1 | β-Parvalbumin | C |
| 305 | f8 | Corn, cereal | Zea mays |  |  | E |
| 306 | f523 | Corn, cereal | Zea mays | rZea m 14 | nsLTP | C |

**Supplementary Table S2: Difference concerning sensitization pattern and fungal colonization of HC, mild to moderate AD and severe AD**

Median and interquartile range of sensitization pattern of ALEX^2®^, ImmunoCAP^®^ as well as of fungal colonization profile in HC, mild to moderate AD and severe AD respectively. Nonparametric statistical analysis using Kruskal-Wallis for comparison of difference in population-median of HC, mild to moderate AD and severe AD together. H-statistic displays whether population-medians are equal, whereas p-values indicate the level of asymptotic significance of comparison of all 3 groups. Post-hoc nonparametric statistical analysis using Mann-Whitney-U Test for pairwise comparison of difference in population-median among the 3 subgroups. U-statistic displays whether population-medians are equal, whereas p-values indicate the level of two-sided asymptotic significance of pairwise comparisons and r-value indicates Pearson’s correlation coefficient for effect size (< 0.3 being small, 0.3-0.5 being medium > 0.5 being large) using Cohen’s classification.

|  | **All 3 subgroups compared** | | | | | **Pairwise comparison of subgroups** | | | | | | | | |
| --- | --- | --- | --- | --- | --- | --- | --- | --- | --- | --- | --- | --- | --- | --- |
|  | **HC** | **mild to moderate AD** | **severe AD** | **Whole study population** | | **HC vs AD mild to moderate** | | | **HC vs AD severe** | | | **mild to moderate AD vs severe AD** | | |
|  | **Distribution** | | | **Kruskal-Wallis** | | **Mann-Whitney-U** | | | **Mann-Whitney-U** | | | **Mann-Whitney-U** | | |
|  | **Median (1-3 quartile)** | | | **H** | **p** | **U** | **p** | **r** | **U** | **p** | **r** | **U** | **p** | **r** |
| Subjects, n | 14 | 9 | 7 | 30 | | 23 | | | 21 | | | 16 | | |
|  | **ALEX^2®^** | | | | | | | | | | | | | |
| tIgE | 0 (0-1) | 4 (3-4) | 4 (4-4) | 24.3 | <.001 | 2 | <0.001 | 0.84 | 14 | <0.001 | 0.77 | 24.5 | 0.20 | 0.32 |
| sIgE |  |  |  |  |  |  |  |  |  |  |  |  |  |  |
| Mala s 5 | 0 (0-0) | 0 (0-0) | 0 (0-1) | 4.1 | 0.13 |  |  |  |  |  |  |  |  |  |
| Mala s 6 | 0 (0-0) | 0 (0-0) | 1 (0-2) | 14.6 | <.001 | 63 | 1 | 0 | 21 | 0.002 | 0.67 | 13.5 | 0.01 | 0.63 |
| Mala s 11 | 0 (0-0) | 0 (0-0) | 0 (0-2) | 4.4 | 0.11 |  |  |  |  |  |  |  |  |  |
| Alt a 1 | 0 (0-0) | 0 (0-0) | 0 (0-0) | 1.9 | 0.39 |  |  |  |  |  |  |  |  |  |
| Alt a 6 | 0 (0-0) | 0 (0-0) | 0 (0-0) | 2.3 | 0.31 |  |  |  |  |  |  |  |  |  |
| Asp f 1 | 0 (0-0) | 0 (0-0) | 0 (0-0) | 0.0 | 1.00 |  |  |  |  |  |  |  |  |  |
| Asp f 3 | 0 (0-0) | 0 (0-0) | 0 (0-0) | 2.3 | 0.31 |  |  |  |  |  |  |  |  |  |
| Asp f 4 | 0 (0-0) | 0 (0-0) | 0 (0-0) | 0.0 | 1.00 |  |  |  |  |  |  |  |  |  |
| Asp f 6 | 0 (0-0) | 0 (0-0) | 0 (0-0) | 1.9 | 0.39 |  |  |  |  |  |  |  |  |  |
| Cla h | 0 (0-0) | 0 (0-0) | 0 (0-1) | 4.1 | 0.13 |  |  |  |  |  |  |  |  |  |
| Cla h 8 | 0 (0-0) | 0 (0-0) | 0 (0-0) | 2.3 | 0.31 |  |  |  |  |  |  |  |  |  |
| Pen ch | 0 (0-0) | 0 (0-0) | 0 (0-1) | 6.8 | 0.03 | 63 | 1 | 0 | 35 | 0.04 | 0.45 | 22.5 | 0.10 | 0.42 |
|  | **ImmunoCAP^®^** | | | | | | | | | | | | | |
| tIgE | 3 (3-4.25) | 6 (5.5-6) | 6 (6-6) | 18.2 | <.001 | 15 | 0.002 | 0.66 | 14 | <0.001 | 0.77 | 24.5 | 0.20 | 0.32 |
| sIgE |  |  |  |  |  |  |  |  |  |  |  |  |  |  |
| m227 | 0 (0-0) | 0 (0-2.5) | 3 (2-4) | 19.6 | <.001 | 35 | 0.01 | 0.78 | 7 | <0.001 | 0.86 | 10.5 | 0.02 | 0.57 |
| m5 | 0 (0-0) | 0 (0-3.1) | 3 (2-3) | 18.4 | <.001 | 45 | 0.09 | 0.36 | 0 | <0.001 | 0.96 | 9 | 0.01 | 0.63 |
|  | **Relative abundance of fungal colonization (%)** | | | | | | | | | | | | | |
| ***Malassezia spp.*** |  |  |  |  |  |  |  |  |  |  |  |  |  |  |
| *glabella* | 91 (83-97) | 94 (92-99) | 89 (73-90) | 5,96 | 0,05 | 46 | 0,28 | 0,27 | 30 | 0,16 | 0,31 | 7 | 0,01 | 0,65 |
| *vertex* | 94 (89-98) | 97 (94-99) | 59 (39-98) | 3,94 | 0,14 | 45 | 0,26 | 0,28 | 29 | 0,14 | 0,33 | 16 | 0,10 | 0,41 |
| *dorsal neck* | 90 (79-96) | 91 (77-96) | 70 (20-94) | 3,03 | 0,22 | 63 | 1,00 | 0,00 | 27 | 0,10 | 0,36 | 18 | 0,15 | 0,36 |
| *antecubital* | 71 (58-84) | 81 (75-95) | 58 (45-81) | 5,43 | 0,07 | 37 | 0,10 | 0,41 | 35 | 0,30 | 0,23 | 11 | 0,03 | 0,54 |
| ***M. sympodialis*** |  |  |  |  |  |  |  |  |  |  |  |  |  |  |
| *glabella* | 1 (0-2) | 0 (0-1) | 0 (0-1) | 3,24 | 0,20 | 46 | 0,28 | 0,27 | 26 | 0,09 | 0,37 | 25 | 0,49 | 0,17 |
| *vertex* | 1 (0-4) | 0 (0-1) | 1 (0-2) | 2,94 | 0,23 | 37 | 0,10 | 0,41 | 42 | 0,60 | 0,11 | 21 | 0,27 | 0,28 |
| *dorsal neck* | 5 (1-13) | 1 (0-6) | 1 (0-2) | 4,95 | 0,08 | 37 | 0,10 | 0,41 | 22 | 0,04 | 0,44 | 30 | 0,87 | 0,04 |
| *antecubital* | 7 (3-13) | 2 (1-4) | 1 (0-3) | 9,87 | 0,01 | 25 | 0,02 | 0,60 | 13 | 0,01 | 0,59 | 24 | 0,43 | 0,20 |
| ***M. globosa*** |  |  |  |  |  |  |  |  |  |  |  |  |  |  |
| *glabella* | 5 (2-10) | 14 (6-29) | 5 (1-51) | 2,92 | 0,23 | 34 | 0,07 | 0,46 | 49 | 1,00 | 0,00 | 23 | 0,37 | 0,23 |
| *vertex* | 5 (2-11) | 5 (2-14) | 4 (2-18) | 0,07 | 0,97 | 59 | 0,80 | 0,06 | 47 | 0,88 | 0,03 | 31 | 0,96 | 0,01 |
| *dorsal neck* | 47 (36-59) | 40 (11-64) | 24 (7-62) | 0,90 | 0,64 | 56 | 0,66 | 0,11 | 37 | 0,37 | 0,20 | 26 | 0,56 | 0,15 |
| *antecubital* | 16 (12-35) | 28 (19-36) | 7 (3-46) | 1,25 | 0,53 | 44 | 0,23 | 0,30 | 39 | 0,46 | 0,16 | 27 | 0,63 | 0,12 |
| ***M. restricta*** |  |  |  |  |  |  |  |  |  |  |  |  |  |  |
| *glabella* | 83 (57-93) | 66 (36-85) | 73 (32-84) | 3,03 | 0,22 | 46 | 0,28 | 0,27 | 26 | 0,09 | 0,37 | 28 | 0,71 | 0,09 |
| *vertex* | 82 (62-94) | 91 (67-94) | 26 (9-88) | 5,29 | 0,07 | 58 | 0,75 | 0,08 | 22 | 0,04 | 0,44 | 12 | 0,04 | 0,52 |
| *dorsal neck* | 33 (27-45) | 38 (17-53) | 12 (7-40) | 3,20 | 0,20 | 57 | 0,71 | 0,09 | 25 | 0,07 | 0,39 | 20 | 0,22 | 0,30 |
| *antecubital* | 41 (30-52) | 41 (22-58) | 26 (15-37) | 5,45 | 0,07 | 63 | 1,00 | 0,00 | 17 | 0,02 | 0,52 | 16 | 0,10 | 0,41 |
| ***Candida spp.*** |  |  |  |  |  |  |  |  |  |  |  |  |  |  |
| *glabella* | 0 (0-1) | 0 (0-0) | 0 (0-4) | 3,88 | 0,14 | 50 | 0,41 | 0,21 | 33 | 0,23 | 0,26 | 12 | 0,04 | 0,52 |
| *vertex* | 0 (0-0) | 0 (0-0) | 0 (0-2) | 6,53 | 0,04 | 44 | 0,22 | 0,31 | 17 | 0,02 | 0,53 | 17 | 0,13 | 0,38 |
| *dorsal neck* | 0 (0-0) | 0 (0-0) | 1(0-1) | 3,16 | 0,21 | 60 | 0,85 | 0,05 | 27,5 | 0,11 | 0,35 | 17 | 0,12 | 0,38 |
| *antecubital* | 0 (0-1) | 0 (0-2) | 1(0-2) | 4,41 | 0,11 | 49 | 0,37 | 0,22 | 23 | 0,05 | 0,43 | 18 | 0,15 | 0,36 |
| ***C. albicans*** |  |  |  |  |  |  |  |  |  |  |  |  |  |  |
| *glabella* | 0 (0-0) | 0 (0-0) | 0 (0-0) | 0,30 | 0,86 | 61,5 | 0,90 | 0,03 | 44 | 0,59 | 0,12 | 29 | 0,70 | 0,10 |
| *vertex* | 0 (0-0) | 0 (0-0) | 0 (0-0) | 2,42 | 0,30 | 57 | 0,63 | 0,12 | 35 | 0,13 | 0,33 | 24,5 | 0,20 | 0,32 |
| *dorsal neck* | 0 (0-0) | 0 (0-0) | 0 (0-0) | 1,34 | 0,51 | 51 | 0,37 | 0,22 | 45 | 0,66 | 0,09 | 24 | 0,33 | 0,24 |
| *antecubital* | 0 (0-0) | 0 (0-0) | 0 (0-0) | 0,90 | 0,64 | 54 | 0,43 | 0,20 | 48 | 0,90 | 0,03 | 26 | 0,44 | 0,19 |

Abbreviations: HC, healthy control, AD, Atopic dermatitis. ALEX^2®^, Allergy Explorer. tIgE, total Immunoglobulin E. sIgE, specific Immunoglobulin E. Mala s 5/6/11, *Malassezia* *sympodialis*. Alt a 1/6, *Alternaria* *alternata*. Asp f 1/3/4/6, *Aspergillus fumigatus*. Cla h/h8, *Cladosporium herbarum*. Pen ch, *Penicilium chrysogenum*. m227 *Malassezia spp. (M. sympodialis, M. globosa, M. restricta)*. m5, *Candida albicans.*

**Supplementary Table S3: Spearman’s correlation of *M. sympodialis* and common inhalative- and food allergens in ALEX^2®^.** *M. sympodialis* sIgE in correlation to the common inhalative- and food allergen sIgE in ALEX^2®^. Results of Spearman’s correlation are illustrated in form of R and p-values.

| **Common name** | **Scientific name** | **Correlation coefficient (R)** | **p-value** |
| --- | --- | --- | --- |
| Alder | Alnus glutinosa | 0,498 | 0,005 |
| Celery | Apium graveolens | 0,534 | 0,002 |
| Silver birch | Betula verrucosa | 0,752 | <,001 |
| Dog | Canis familiaris | 0,635 | <,001 |
| Hazel | Corylus avellana | 0,488 | 0,006 |
| Hazelnut | Corylus avellana | 0,516 | 0,004 |
| Bermuda grass | Cynodon dactylon | 0,669 | <,001 |
| Carott | Daucus carota | 0,51 | 0,004 |
| American house dust mite | Dermatophagoides farinae | 0,565 | 0,001 |
| European house dust mite | Dermatophagoides pteronyssinus | 0,75 | <,001 |
| Horse | Equus caballus | 0,505 | 0,004 |
| Beech | Fagus sylvatica | 0,457 | 0,011 |
| Cat | Felis domesticus | 0,439 | 0,015 |
| Ash | Fraxinus excelsior | 0,789 | <,001 |
| Lepidoglyphus destructor | Lepidoglyphus destructor | 0,596 | <,001 |
| Perennial ryegrass | Lolium perenne | 0,653 | <,001 |
| Mouse | Mus musculus | 0,629 | <,001 |
| Olive | Olea Europaea | 0,779 | <,001 |
| Rabbit | Oryctolagus cuniculus | 0,482 | 0,007 |
| Timothy | Phleum pratense | 0,752 | <,001 |
| Rye | Secale cereale | 0,549 | 0,002 |

**Supplementary Figures**


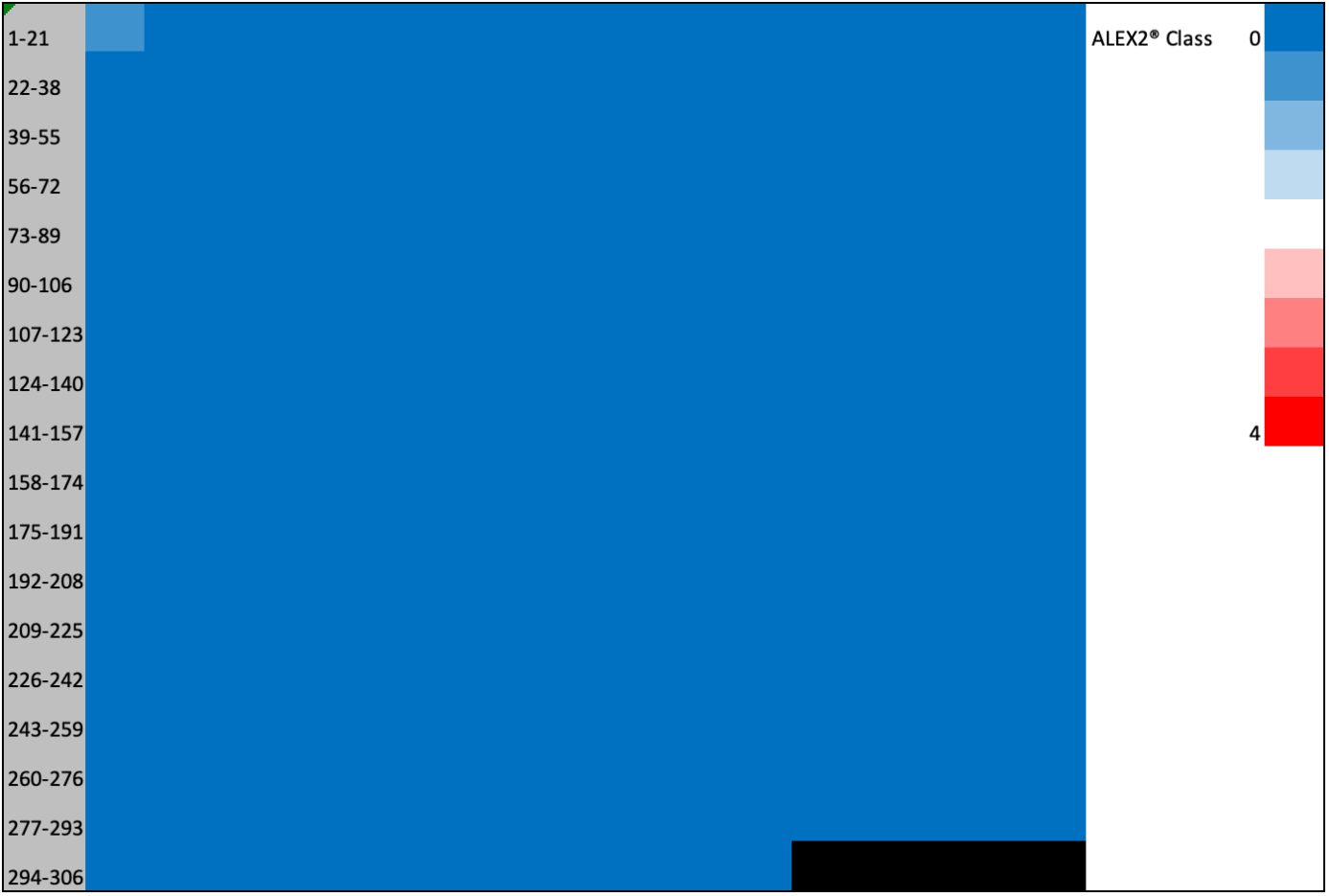


**Supplementary Figure S1: Heat map of sensitization class in ALEX^2®^ in HC**

Means of tIgE (N°1, upper left field) and sIgE sensitization towards the whole ALEX^2®^ macroarray (N° 2-306) of the whole subgroup. From dark blue for class 0 over white for class 2 towards dark red for class 4. Black fields represent empty space on macroarray. Means being rounded to the nearest 0.5 with 0.25 being rounded up.


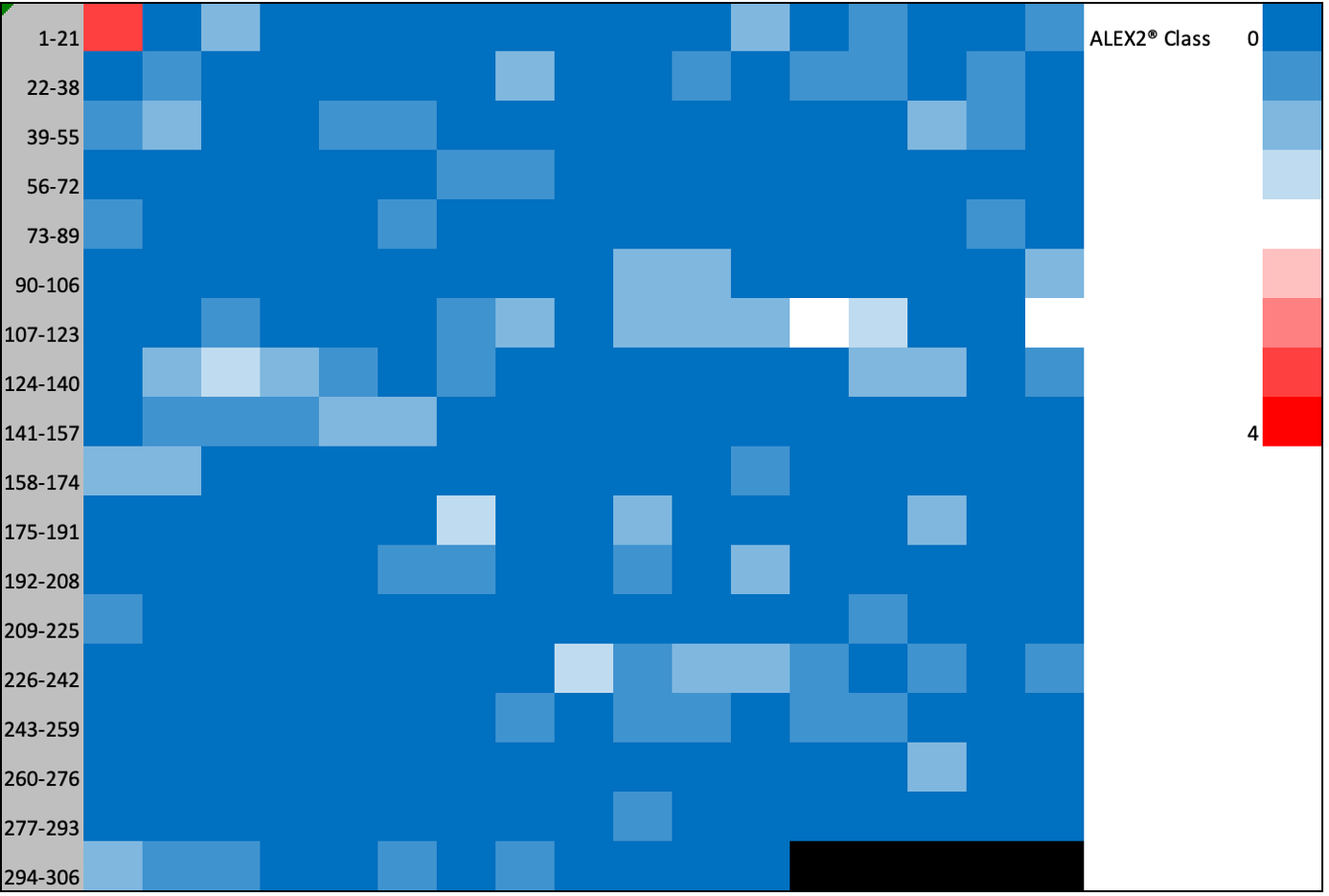


**Supplementary Figure S2: Heat map of sensitization class in ALEX^2®^ in mild to moderate AD**

Means of tIgE (N°1, upper left field) and sIgE sensitization towards the whole ALEX^2®^ macroarray (N° 2-306) of the whole subgroup. From dark blue for class 0 over white for class 2 towards dark red for class 4. Black fields represent empty space on macroarray. Means being rounded to the nearest 0.5 with 0.25 being rounded up.


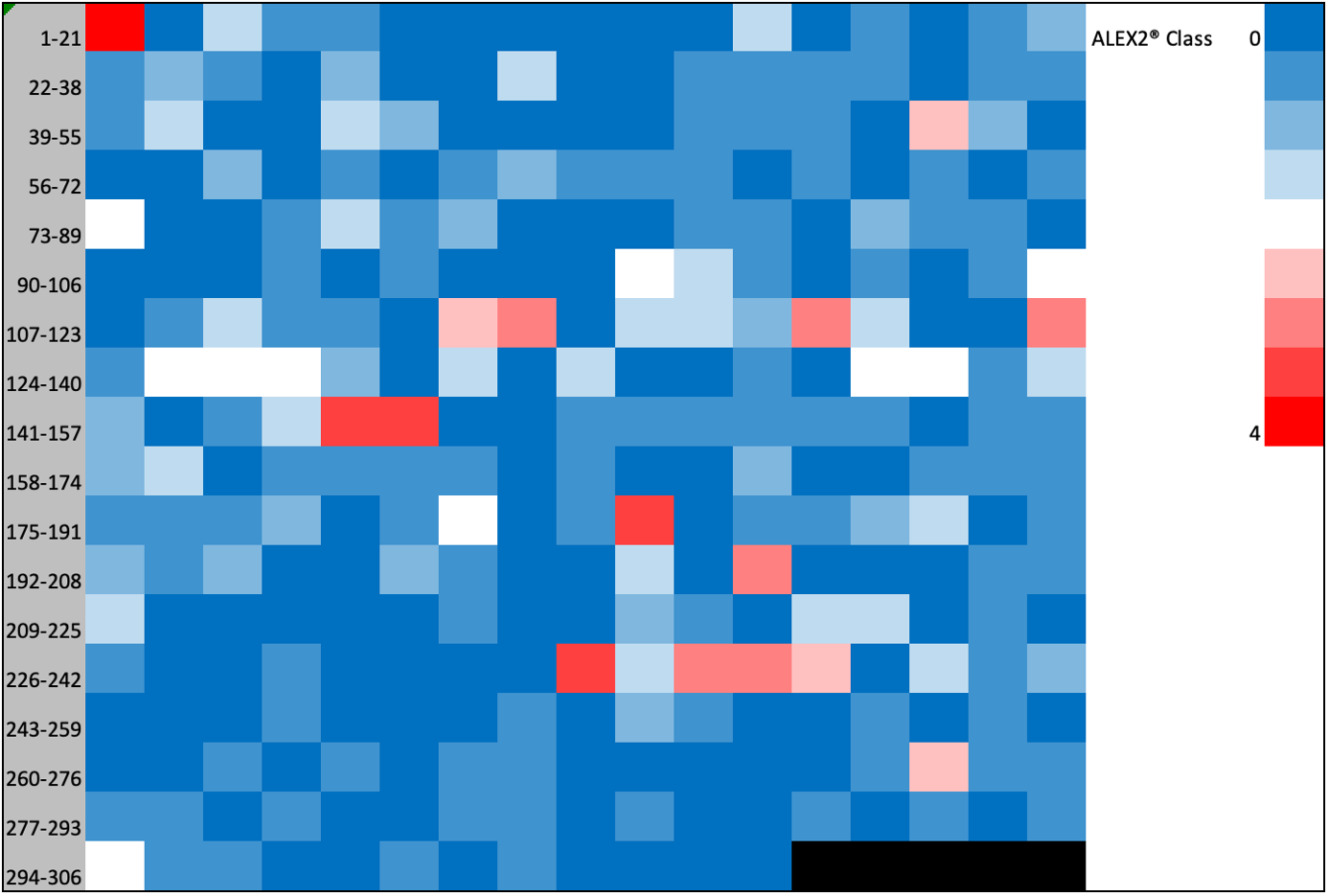


**Supplementary Figure S3: Heat map of sensitization class in ALEX^2®^ in severe AD**

Means of tIgE (N°1, upper left field) and sIgE sensitization towards the whole ALEX^2®^ macroarray (N° 2-306) of the whole subgroup. From dark blue for class 0 over white for class 2 towards dark red for class 4. Black fields represent empty space on macroarray. Means being rounded to the nearest 0.5 with 0.25 being rounded up.


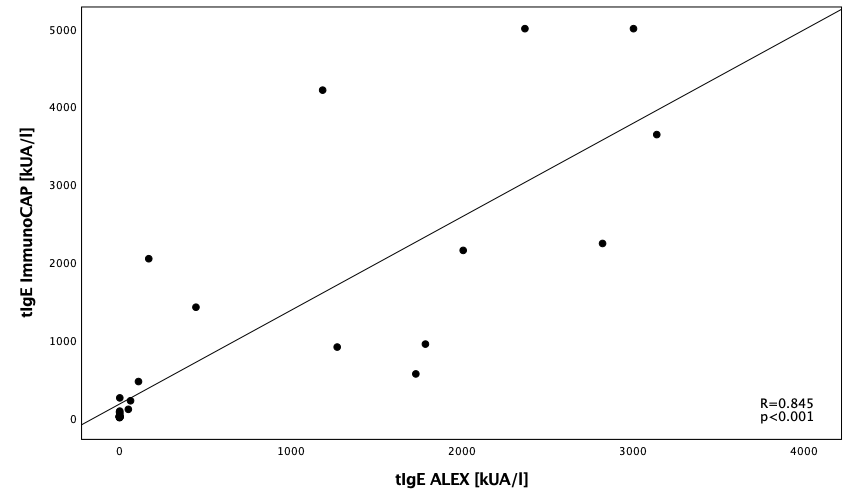


**Supplementary Figure S4: Spearman’s correlation of tIgE using ImmunoCAP^®^** **and ALEX^2®^.** ALEX^2®^ tIgE in correlation to ImmunoCAP^®^ tIgE displayed as Class. Results of Spearman’s correlation are illustrated in form of R and p-values.


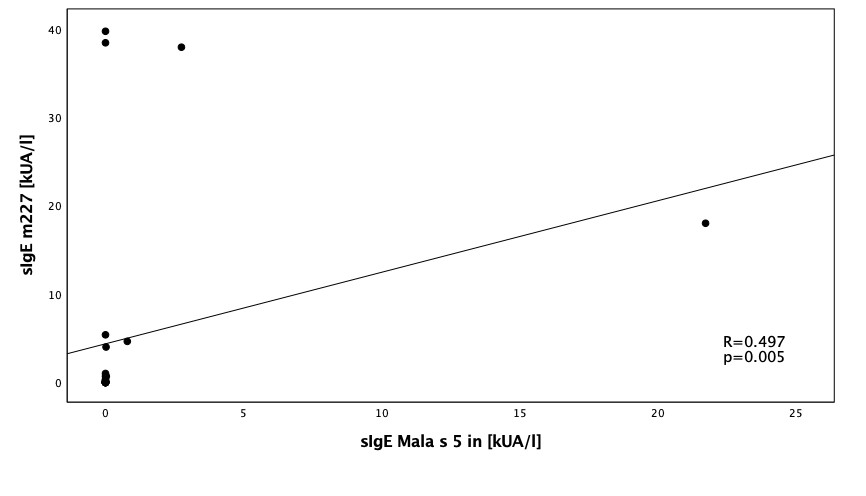
**A**


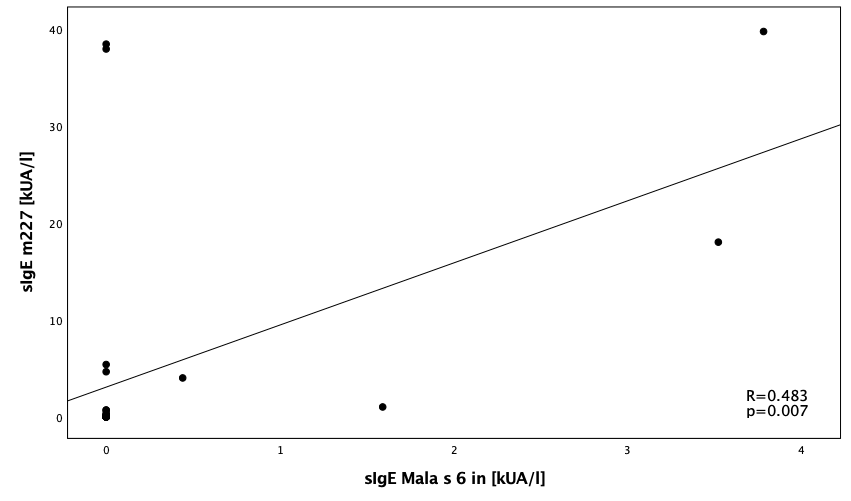


**B**


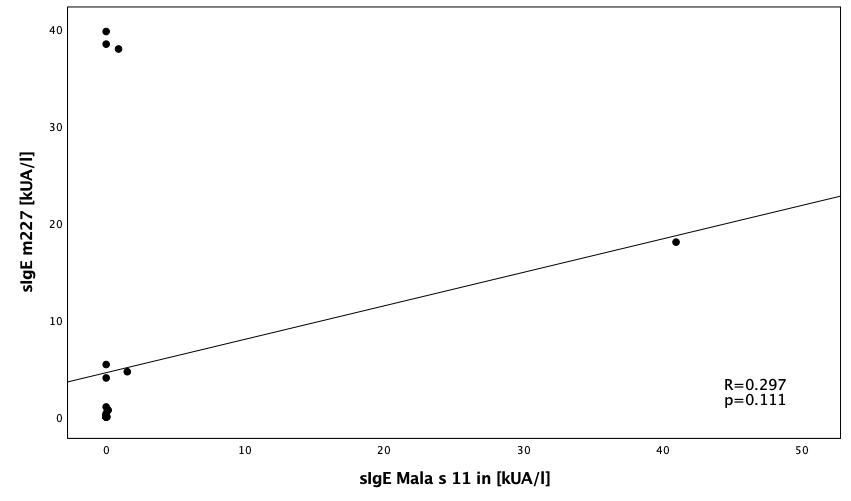


**C**

**Supplementary Figure S5: Spearman’s correlation of specific sensitization towards *Malassezia* spp. allergens among the whole study population using ImmunoCAP^®^ and ALEX^2®^.** sIgE m277 ImmunoCAP^®^ containing allergens of M. sympodialis, M. globosa and M. restricta and is shown in correlation towards sIgE of M. sympodialis ALEX^2®^ (A) Mala s 5, (B) Mala s 6 and (C) Mala s 11respectively. Results of Spearman’s correlation are illustrated in form of R and p-values.

**
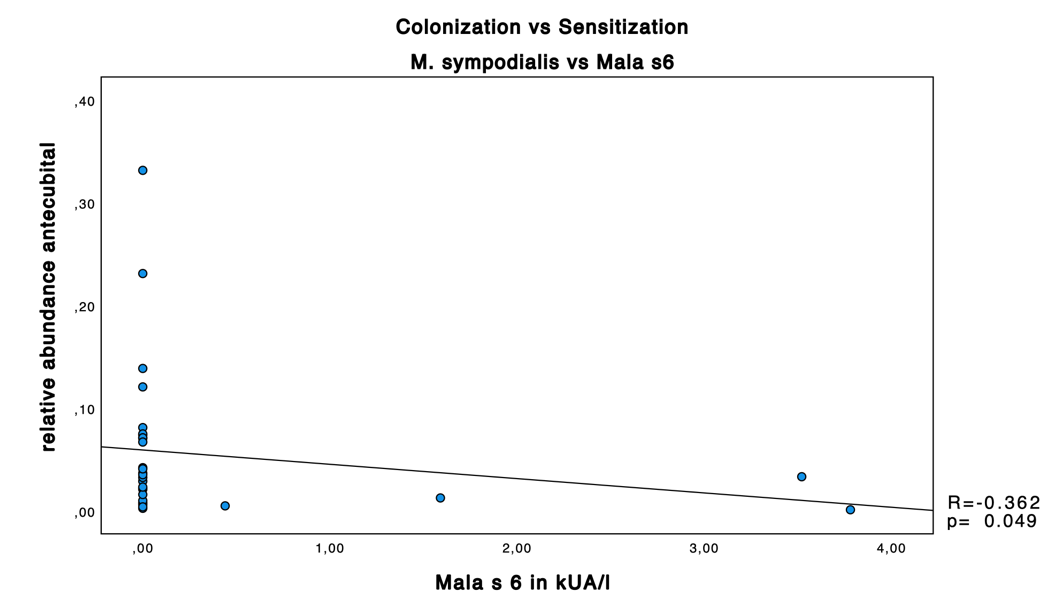
A**

**
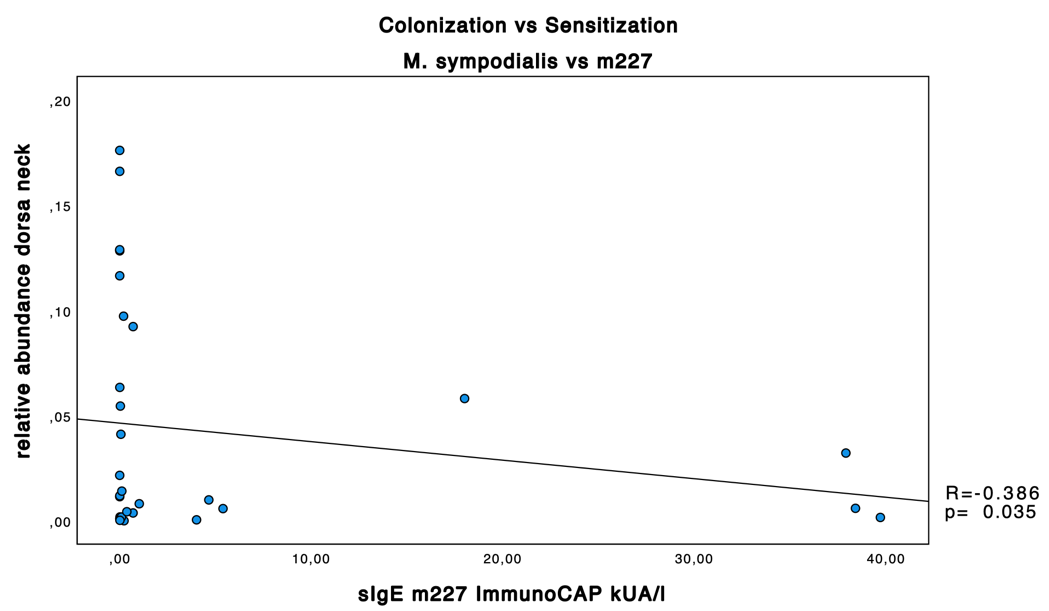
**

**B**

**
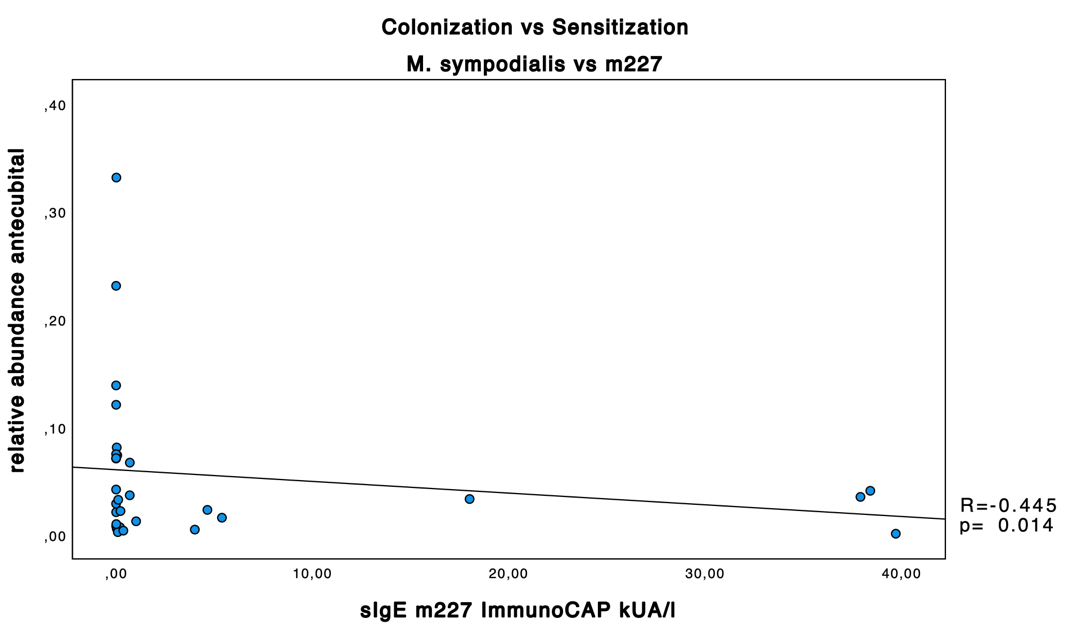
**

**C**

**
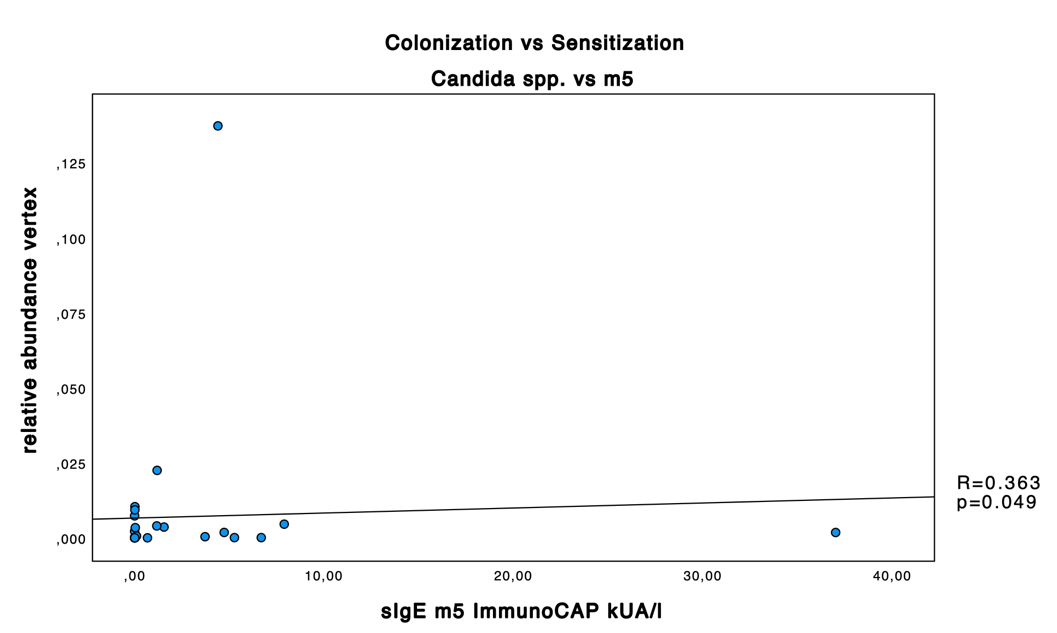
D**

**Supplementary Figure S6 Correlation of skin colonization with M. sympodialis and Candida spp. and sensitization towards Malassezia spp. and C. albicans**

Correlation between relative abundance of *M. sympodialis* and (A) sensitization towards *M. sympodialis* using ALEX^2®^, (B&C) sensitization towards *M. sympodialis, M. globosa and M. restricta* using ImmunoCAP^®^. (D) Correlation between relative abundance of Candida spp. and sensitization towards C. albicans using ImmunoCAP^®^. Results of Spearman’s correlation are illustrated in form of R and p-values.
